# Analysis of *Magnaporthe oryzae* Genome Reveals a Fungal Effector, Which Is Able to Induce Resistance Response in Transgenic Rice Line Containing Resistance Gene, *Pi54*

**DOI:** 10.3389/fpls.2016.01140

**Published:** 2016-08-08

**Authors:** Soham Ray, Pankaj K. Singh, Deepak K. Gupta, Ajay K. Mahato, Chiranjib Sarkar, Rajeev Rathour, Nagendra K. Singh, Tilak R. Sharma

**Affiliations:** ^1^National Research Centre on Plant Biotechnology, Pusa CampusNew Delhi, India; ^2^Chaudhary Sarwan Kumar Himachal Pradesh Agricultural UniversityPalampur, India

**Keywords:** avirulence gene, protein–protein interaction, yeast-two-hybrid analysis, functional complementation, rice blast, *AvrPi54*

## Abstract

Rice blast caused by *Magnaporthe oryzae* is one of the most important diseases of rice. *Pi54*, a rice gene that imparts resistance to *M. oryzae* isolates prevalent in India, was already cloned but its avirulent counterpart in the pathogen was not known. After decoding the whole genome of an *avirulent* isolate of *M. oryzae*, we predicted 11440 protein coding genes and then identified four candidate effector proteins which are exclusively expressed in the infectious structure, appresoria. *In silico* protein modeling followed by interaction analysis between Pi54 protein model and selected four candidate effector proteins models revealed that Mo-01947_9 protein model encoded by a gene located at chromosome 4 of *M. oryzae*, interacted best at the Leucine Rich Repeat domain of Pi54 protein model. Yeast-two-hybrid analysis showed that Mo-01947_9 protein physically interacts with Pi54 protein. *Nicotiana benthamiana* leaf infiltration assay confirmed induction of hypersensitive response in the presence of *Pi54* gene in a heterologous system. Genetic complementation test also proved that Mo-01947_9 protein induces avirulence response in the pathogen in presence of *Pi54* gene. Here, we report identification and cloning of a new fungal effector gene which interacts with blast resistance gene *Pi54* in rice.

## Introduction

Rice is one of the most important cereal crops and staple food of about 2.7 billion people in the world (Fairhurst and Dobermann, 2002). The production of rice is seriously affected by several biotic and abiotic stresses. Among all the biotic stresses, rice blast caused by *Magnaporthe oryzae* is one of the most devastating diseases of rice that causes tremendous yield loss every year worldwide (Ou, 1985; Wang and Valent, 2009; Wilson and Talbot, 2009; Fisher et al., 2012). The rice crop is vulnerable to this pathogen from seedlings to adult plant stages^1^. *M*. *oryzae* employs a series of developmental and metabolic pathways during infection process after the conidia land on the waxy leaf surface, until production of sporulating lesions. Rice and *M. oryzae* constitute an ideal pathosystem to study plant–pathogen interaction (Valent and Chumley, 1991; Xu et al., 2006) conforming to a typical gene-for-gene system (Flor, 1971) where an avirulence (*Avr*) gene product from the pathogen interacts with the corresponding resistance (*R*) gene product of the host. When both *R-* gene and *Av-* gene are present in dominant form, their protein products interact with each other at the cellular level to give rise to resistance response via hypersensitive cell death pathway. Besides direct interaction between R- and Avr- proteins as proposed by classical receptor-ligand model, indirect interaction between the two is also reported. According to guard hypothesis (Van der Biezen and Jones, 1998; Dangl and Jones, 2001), *R*- gene product functions as a ‘guard’ for virulence target (operative target) in the host cell known as ‘guardee.’ The Avr- protein interacts with ‘guardee’ and tries to manipulate it in order to make the plant susceptible; whereas the R- protein, functioning as a guard, can identify the structural deviation in guardee caused by effector binding and can bind to such Guardee-effector complexes to initiate defense response. In such a situation, the effector will be regarded as Avr- protein. But the concept of guard hypothesis also has an inherent evolutionary conflict as it implies that the in absence of functional *R*- gene natural selection would provide selective advantage to the effector targets (guardees) that escape recognition by the effector while in presence of functional *R*- gene it will render advantage to the effector targets (guardees) that enhance recognition. Decoy model (Zhou et al., 1995; Zipfel and Rathjen, 2008), however, can relax this evolutionary conflict imparted on ‘guardee’ by evolution of some host molecules termed as ‘decoy’ which are the molecular mimic of the guardee (operative target) but do not have any role in disease development and hence do not aid in pathogen fitness in absence of functional *R*- gene. But these decoys can have high affinity toward pathogen effector and the decoy-effector complex can initiate resistance response in presence of functional *R*- gene. Here it is important to mention that avirulence (*Avr*) proteins also come under effector protein involved in pathogenicity (Rouxel and Balesdent, 2010).

Majority of fungal *Avr* genes have been cloned from those fungi that colonize at intercellular spaces in the plant tissue. Most of these genes were cloned by identifying extracellular proteins that elicit plant cell necrosis in a race-specific manner (Lague and De Wit, 1998). For example, *Avr9* gene cloned from tomato pathogenic fungi *Cladosporium fulvum* is an earliest example of avirulence gene cloning which was cloned by using similar method (Van Kan et al., 1991). However, *M. oryzae* is a hemibiotroph that colonizes in the intracellular spaces of the plant and hence its *Avr* genes cannot be cloned by reverse genetic approaches because of the difficulties in identifying the Avr protein inside the host cell. Therefore, map-based cloning was the only strategy available to clone *Avr* genes of *M. oryzae*. As the natural isolates of *M. oryzae* are female sterile, it is difficult to develop a mapping population, which is a prerequisite for map-based gene cloning. For this purpose, a fertility restorer gene has to be transferred from another *Magnaporthe grisea* (pathogenic to grasses other than rice) isolate, which is non-pathogenic to rice, through backcross to restore fertility before carrying out map-based cloning. Using this approach, seven *Avr* genes, *ACE-*1 (Böhnert et al., 2004), *PWL1, PWL3, PWL4* (Kang et al., 1995), *PWL2* (Sweigard et al., 1995), *Avr Pi-ta* (Orbach et al., 2000), *Avr1-Co39* (Farman and Leong, 1998), *AvrPiz-t* (Li et al., 2009), and *AvrPib* (Zhang et al., 2015), have been cloned from *M. oryzae* till date. With the availability of the whole genome sequence of *M. oryzae* (strain 70-15) in the public domain and the advent of high throughput next generation sequencing techniques, genome-wide *Avr* gene searching followed by association genetic studies have come up as an alternate approach to clone *Avr* genes from *M. oryzae.* Following such a strategy, three new *Avr* genes, *Avr-Pia* (Yoshida et al., 2009), *Avr-Pii* (Yoshida et al., 2009), and *Avr-Pik/km/kp* (Yoshida et al., 2009), have recently been cloned but their functions have not been validated *in vivo*.

It has been reported that a dominant blast resistance gene *Pi54* (previously known as *Pi-k^h^*), present in indica rice line ‘Tetep’ exhibits resistance to *M*. *oryzae* in India (Sharma et al., 2005b). This gene has been cloned and functionally validated (Sharma et al., 2005a; Rai et al., 2011). However, its counterpart *Avr* gene (*AvrPi54*) in the pathogen has not been cloned till date. As the whole genome sequence (41.7 Mbp) of *M. oryzae* (Dean et al., 2005) is now available in the public domain^2^), its analysis will provide insight into genome structure and might help in cloning of effector genes (including *Avr* genes) by using various computational tools such as homology-based protein modeling and molecular docking of *R* and *Avr* proteins. This approach has been widely applied and is successful in the field of *in silico* drug discovery and also used in R–Avr interaction studies (Wang et al., 2007; Ehrenfeld et al., 2008). Most importantly, this strategy has the potential to reduce the time needed for cloning of an effector gene by many folds when its protein target in the host is known. Pi54, being a resistance protein (or guard), must interact physically with AvrPi54 protein according to a classical receptor–ligand interaction or with the effector-guardee complex. We postulated our null hypothesis that Pi54 protein and the AvrPi54 effector protein are involved in classical receptor–ligand interaction. Based on this hypothesis following objectives for the study were formulated: (i) whole genome sequencing of an avirulent strain of *M. oryzae* and *in silico* identification of potential candidates for *AvrPi54* gene, (ii) construction of 3D protein models of selected *AvrPi54* candidates and *in silico* protein–protein interaction analysis between *Pi54* and candidate *AvrPi54* genes products (iii) *in vitro* confirmation of physical interaction between *Pi54* and candidate *AvrPi54* gene(s) by Yeast-2-Hybrid (Y2H) analysis, and (iv) *in vivo* detection of avirulence function of candidate effector protein in the presence of *Pi54* gene.

## Results

### Identification of *Avr* Genes in the *M. oryzae* Genome

We sequenced the whole genome of an avirulent isolate RML-29 of *M. oryzae* using 454 FLX pyrosequencing method. RML-29, which is avirulent on *Pi54* gene, is expected to carry the *AvrPi54* gene in its genome. The sequence assembly included 1357128 number of high quality (>Phred 20) sequence reads assembled in 2300 contigs. The N50 and largest assembled contig length of RML-29 genome were calculated as 60 kb and 381 kb, respectively (**Table 1**). We also filtered out 400 unique contigs specific to RML-29 genome, which did not show any significant alignment against the reference genome of *M. oryzae* isolate 70-15, which is available in the public domain (Dean et al., 2005).

**Table 1:** Structural features of *M. oryzae* isolate RML-29 whole genome sequence.

Total number of sequenced reads	1429811
Total length of sequence read	545.18 Mb
Coverage of the genome	13.29X
Total number of contigs	2300
Total size of contigs	37.20 Mb
Largest contigs	381875 bps
G+C Content	51.76%
Average contig size	16446 bps
N50 contigs size	60619 bps
Total number of mapped contigs	1900
Total number of unmapped contigs	400
The size of RML-29 DNA regions unmapped to 70-15 contigs	2.36 Mb (6.34%)
The size of RML-29 DNA regions without match to70-15 raw read sequences	0.14 Mb
Number of predicted protein coding genes in the whole genome	11440


**FIGURE 1:**
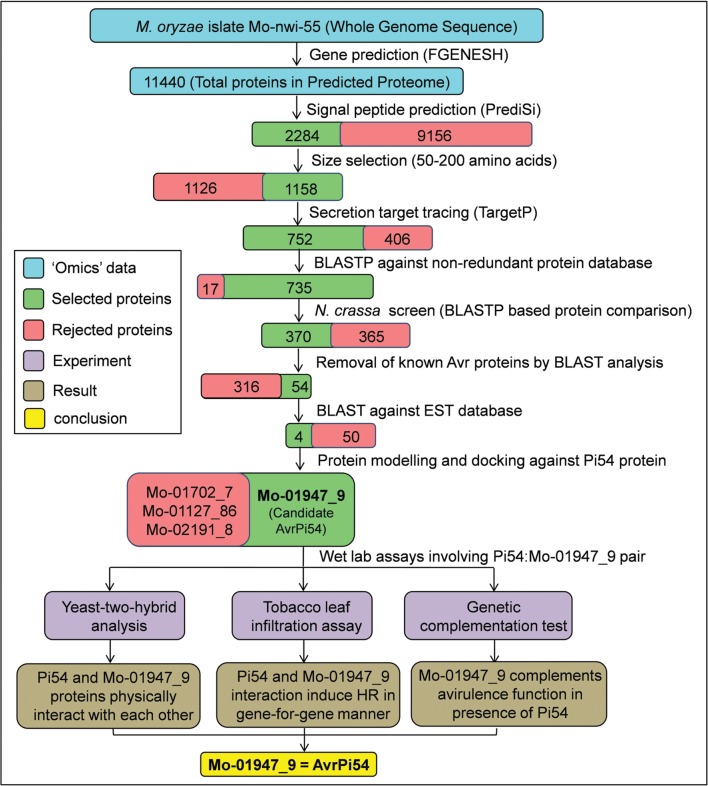
**Flow diagram summarizing the methodology for identification of *AvrPi54* gene.** Candidate AvrPi54 was identified *in silico* utilizing the whole genome sequence of *M. oryzae* isolate Mo-nwi-55 and was further validated through wet lab experiments.

**FIGURE 2:**
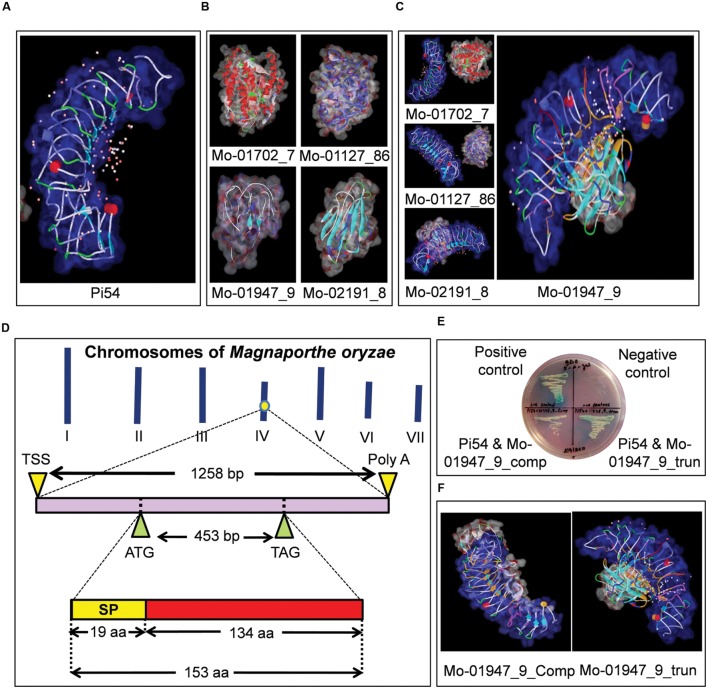
**Identification of AvrPi54 gene in *Magnaporthe oryzae* genome which encodes a secreted protein, that interacts with blast resistance protein Pi54.** 3-D models of Pi54 protein **(A)** and candidate AvrPi54 proteins **(B)** generated by MODELER software inbuilt in Accelrys Discovery Studio 2.1. *In silico* interaction study between Pi54 protein and the four selected candidate AvrPi54 proteins by docking analysis (carried out in ZDOCK software inbuilt in Accelrys Discovery Studio 2.1) revealed, Mo-01947_9 protein is the best candidate to be qualified as AvrPi54 protein as it showed best interaction with Pi54 protein. The Pi54 protein is shown in blue in all the cases and the interacting amino acids of both Pi54 and candidate AvrPi54 proteins are shown in orange **(C)**. *In silico* mapping showed that the candidate AvrPi54 (Mo-01947_9) is a unique gene located at chromosome 4. It is a 1258 bp long gene containing a single unfragmented ORF of 462 bp. It encodes a protein consisting of 153 amino acids among which first 19 amino acids from the N-terminal end encodes for a signal peptide (SP) needed for secretion **(D)**. Yeast-2-Hybrid analysis confirmed the interaction between Pi54 protein and candidate AvrPi54 protein (Mo-01947_9) *in vitro*. Interaction potential was checked for both full length (Mo-01947_9_comp) as well as SP truncated version (Mo-01947_9_trun) of candidate AvrPi54 protein with the Pi54 protein. Better growth of yeast cells clearly suggested better interaction potential of SP truncated version (Mo-01947_9_trun) of candidate AvrPi54 protein with Pi54 protein compared to its full length (Mo-01947_9_comp) version **(E)**. *In silico* docking analysis also supported this fact as the pose and strength of interaction was better in case of SP truncated version of AvrPi54 protein (Mo-01947_9_trun) compared to the full length version of AvrPi54 protein (Mo-01947_9_comp) **(F)**.

In the whole genome sequence of *M. oryzae* isolate RML-29, we began our search to identify probable *AvrPi54* candidates *in silico* following multiple steps (**Figure 1**). We hypothesized that Pi54 protein (**Figure 2A**) and AvrPi54 protein should interact physically with each other to induce resistance response following typical gene-for-gene hypothesis. We predicted 11440 protein coding genes (which encode protein having ≥50 amino acids and the encoded protein begins with methionine) in the whole genome of *M. oryzae* strain RML-29.

As the R–Avr protein interactions take place inside the host cell, the AvrPi54 protein, similar to other effector proteins, must be secreted outside of the host cell. Therefore, to determine the secreted proteins that are targeted to exocytic pathways, the presence of signal peptide (SP)/transit peptide (TP) were analyzed in the predicted 11440 proteins using PrediSi software^3^. These SP/TP are needed for sorting of proteins to different organelles or for secretion. A total 2284 proteins were found to contain N-terminal SP/TP and therefore are expected to be capable of sorting or secretion. These 2284 proteins were passed through a size exclusion screen where proteins having a size of up to 330 amino acids (size of the Pi54 protein) were only retained based on the fact that all the *Avr* proteins reported so far and known to follow gene-for-gene system, are smaller in size than their counterpart *R* protein. Using this size exclusion screen, 1158 proteins were further identified. Out of these 1158, not all the proteins having N-terminal SP are directed toward secretory pathways. The subset of proteins that are destined for secretion can further be identified by their specific SP pattern using TargetP software^4^. A subset containing 752 proteins was found to have secretion signals. Removing the already annotated proteins from this subset by Basic Local Alignment Search Tool for Protein (BLAST P) analysis against non-redundant protein database, 735 unique proteins were retained. These 735 proteins were BLAST searched against *Neurospora crassa* proteome, which is the closest non-pathogenic relative of *M. oryzae* (Dean et al., 2005; Kasuga et al., 2009). Following this analysis, 370 proteins were obtained that were uniquely present in *M. oryzae* but absent in *N. crasssa* and therefore might be associated with the pathogenicity of *M. oryzae*. As this set of 370 proteins will also contain other *Avr* genes, known *Avr* genes and their close homologs were removed, which ultimately reduced the search space to 54 proteins (**Supplementary Table S1**). All these 54 proteins were found to be hypothetical proteins based on their annotation by BLAST P against non-redundant protein database available at National Center for Biotechnology Information (NCBI; **Supplementary Table S2**). It has been reported previously that most of the *Avr* genes of *M. oryzae* destined for secretion are expressed either constitutively or in appressoria (Khang et al., 2010). We BLAST searched the identified 54 proteins against EST library (est_others) available in the NCBI and found that four proteins (Mo-01702_7, Mo-01127_86, Mo-01947_9, and Mo-02191_8) among these were expressed in appressoria of *M. oryzae.* Various features of these selected candidates proteins are given in **Supplementary Table S3**.

### Protein Modeling of *Avr* Genes

*In silico* mapping and annotation of the four selected candidates revealed that the candidate genes, Mo-01702_7, Mo-01127_86, Mo-01947_9, and Mo-02191_8 were located at chromosome 7, 2, 4, and 1, respectively, of *M. oryzae* (**Supplementary Table S3**). All these genes encoded small secreted proteins having size range between 103 and 267 amino acids and can be identified by the presence of N-terminal SP of variable sizes (**Supplementary Table S3**). The mature proteins (protein after SP truncation) show high degree of variability between them with respect to their secondary and tertiary structures. For example, the protein Mo-01702_7 contained 28 α-helices but no β-sheet, whereas Mo-01947_9 contained no α-helix but six β-sheets (**Figure 2B**). The structural details of the modeled candidate proteins along with the results of Ramachandran plot analysis is given in **Supplementary Table S4**.

### *In silico* Interaction between Blast Resistance Gene *Pi54* and Candidate *AvrPi54* Genes

Docking results of the Pi54 protein (encoded by rice blast resistance gene *Pi54*) and the protein products of the candidate *Avr* genes generated in the present study showed that only one candidate Avr protein, that is, Mo-01947_9 showed physical interaction at the Leucine Rich Repeat (LRR) domain of Pi54 protein model with Atomic Contact Energy (ACE) of -139.17 kcal/mol and calculated binding energy of -313.54 kcal/mol. The other three candidates (Mo-02191_8, Mo-01702_7, and Mo-1127_86) did not show any physical interaction with the Pi54 protein as suggested by the positive ACE values of interactions (224.78, 424.98, and 490.16, respectively; **Figure 2C**, **Supplementary Table S5**). Among the amino acids of Pi54 protein involved in interaction with Mo-01947_9, it includes 10 amino acid residues (L221, E222, N223, L224, S225, I226, S227, F228, E231, and K249) of LRR domain. This is particularly relevant as the LRR domain of R- proteins is the site of interaction with the Avr- proteins (Jia et al., 2000; Ravensdale et al., 2012). BLAST analysis using the nucleotide sequence of the gene encoding Mo-01947_9 protein, against the *M. oryzae* genome revealed that it is a unique gene of 1258 bp located at chromosome 4 (1023494 bp to 1024751 bp on the minus strand) and consists of a single exon of 462 bp. It encodes a protein containing 153 amino acids with an expected molecular weight of 15.78 kD. Further, it is predicted to be a secreted protein because of the presence of a 19 amino acid-long SP at the N-terminal region (**Figure 2D**). The predicted secondary structure reveals the presence of six β-sheets, whereas α-helices were absent in the mature SP truncated protein. The Ramachandran plot (**Supplementary Figure S1**) of the candidate AvrPi54 protein model shows that out of 153 amino acids of the full length protein, 95.4% were in the favored region, 2.8% were in the allowed region, whereas the remaining 1.9% were in the disallowed region. The gene sequence from *M. oryzae* isolate RML-29 has been deposited in European Nucleotide Archive (ENA) of European Molecular Biology Laboratory (EMBL) Nucleotide Sequence Database under the accession number HF545677. We also docked the candidate AvrPi54 protein (Mo-01947_9) model against the protein model encoded by the susceptible allele of *Pi54* gene from cultivar Taipei 309 (Pi54_TP-309). Compared to the protein encoded by resistant *Pi54* allele (Pi54_Tetep), which is of 330 amino acids in length, Pi54_TP-309 is a 276 amino acid-long protein having N-terminal deletion (**Supplementary Figure S4A**). Expectedly, candidate AvrPi54 protein, Mo-01947_9, did not show any physical interaction with Pi54_TP309 (**Supplementary Figure S4B**) abiding Flor’s hypothesis.

### Interaction between Pi54 and Candidate AvrPi54 (Mo-01947_9) Proteins and Induction of Hypersensitive Response (HR)

Yeast-two-hybrid (Y2H) analysis was performed to confirm the *in silico* interaction between the Pi54 protein and candidate AvrPi54 protein (Mo-01947_9). Both the full length protein and SP truncated protein were used in this study. The Gal4 DNA binding domain (BD) fusion construct of Pi54 (BD::Pi54) with the Activation Domain (AD) fusion constructs of complete candidate AvrPi54 (AD::AvrPi54_comp) and the SP truncated candidate AvrPi54 (AD::AvrPi54_trunc), respectively, were tested for activation of expression from the GAL4 upstream activating sequence in an Y2H assay. Coexpression of BD::Pi54 and AD::AvrPi54_comp fusion proteins as well as BD::Pi54 and AD::AvrPi54_trunc fusion proteins activated three reporter genes, *HIS3*, *ADE2*, and α*-galactosidase* simultaneously thus confirming that physical interaction did occur *in vitro* between the proteins under study (**Figure 2E**). Interaction studies of reverse fusion of Pi54 (AD::Pi54) and AvrPi54 (BD::AvrPi54_comp and BD::AvrPi54_trunc) proteins also confirmed the authenticity of the physical interaction between the Pi54 and candidate AvrPi54 proteins (**Supplementary Figure S2**). But, the reduction of interaction potential, between Pi54 protein and candidate AvrPi54 protein, because of the presence of N terminal SP was also observed *in silico*. Pi54:AvrPi54_trunc interaction was found to be stronger than Pi54:AvrPi54_comp interaction (**Figure 2F**) indicating that truncation of SP (which is the common phenomenon during secretion of proteins *in vivo*) makes the candidate AvrPi54 protein more competent for interaction with Pi54 protein. It was also found that SP truncated candidate AvrPi54 protein model interacted with more amino acid residues of Pi54 protein model compared to full length AvrPi54 protein model (**Supplementary Table S6**). Our results of Y2H analysis therefore showed that Pi54 may function as a receptor protein in the host that can recognize candidate AvrPi54 protein secreted by *M. oryzae* during host invasion.

**FIGURE 3:**
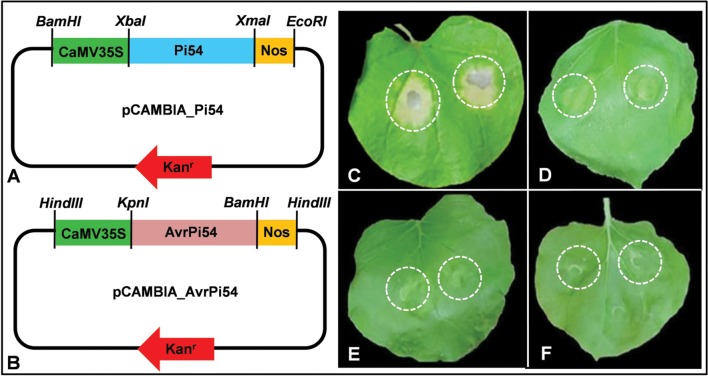
**Tobacco leaf infiltration assay demonstrates HR induction in response to direct physical interaction between Pi54 and candidate AvrPi54 proteins.** Line diagrams represent Pi54 expression module **(A)** and AvrPi54 expression module **(B)** used for agroinfiltration. HR symptoms were observed when Pi54 and AvrPi54 expression modules were co-infiltrated in tobacco leaf **(C)**. Neither AvrPi54 expression module nor Pi54 expression module produced HR when infiltrated individually (**D,E**, respectively). Empty pCAMBIA vector (negative control) also did not induce HR upon infiltration **(F)**. The sites of agroinfiltrations in the tobacco leaves are marked by white dashed circles.

Further, to understand if this interaction between Pi54 and candidate AvrPi54 (Mo-01947_9) proteins, is necessary and sufficient to induce HR or there are other players involved in this process, we performed *N. benthamiana* leaf infiltration assay. Using agroinfiltration, *Pi54* and candidate *AvrPi54* genes were transiently co-expressed in the leaves of *N. benthamiana* which induced strong HR. However, HR was not detected when these two genes were expressed individually (**Figure 3**; **Supplementary Figure S3**; **Supplementary Table S7**). Hence, the obtained results clearly suggest that physical interaction between Pi54 and candidate AvrPi54, proteins is necessary and sufficient to induce HR.

### Genetic Complementation Test of Candidate *AvrPi54* Gene

**FIGURE 4:**
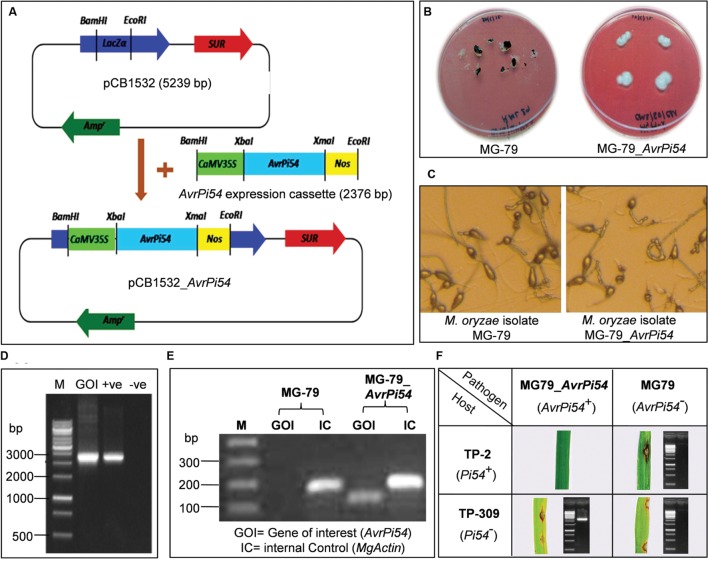
**Validation of avirulence function of *AvrPi54* gene by complementation test.** *AvrPi54* expression cassette (2376 bp) was cloned in the *Bam*HI-*Eco*RI site of fungal transformation vector pCB1532 (5239 bp) to produce pCB1532_AvrPi54 **(A)**. After 10 days the non-transgenic control (MG-79) showed symptoms of withering but the transgenic fungus grew (MG-79_*AvrPi54*) normally **(B)**. Microscopic analysis revealed no significant morphological differences between transgenic and non-transgenic isolates **(C)**. To further confirm the success of transformation, *AvrPi54* expression cassette (EC, 2376 bp) was amplified from genomic DNA of transgenic isolate MG-79_*AvrPi54* (). pCB1532_*AvrPi54* plasmid DNA (+ve) and genomic DNA of non-transgenic isolate MG-79 (-ve) were used as template used as templates which served as positive and negative controls, respectively **(D)**. Semiquantitative RT-PCR analysis shows the expression of the *AvrPi54* gene in transgenic isolate (102 bp target amplicon) but not in non-transgenic. *M. oryzae Actin* gene (housekeeping gene) was used as internal control (175 bp amplicon) for this experiment **(E)**. Complementation of avirulence function was tested by inoculating 15 days old seedlings of TP-309 (rice line that do not contain *Pi54* gene) and TP-2 (transgenic rice line that contain *Pi54* gene in TP-309 background) with *M. oryzae* strains MG-79 and MG-79_AvrPi54 **(F)**.

Complementation of avirulence function of the cloned candidate *AvrPi54* gene (Mo-01947_9) was studied by standard pathogen challenge experiments against susceptible rice line Taipei 309 (TP-309) as well as resistant transgenic line TP-2 (which contains blast resistance gene *Pi54* in TP-309 background). These two lines were challenged with virulent isolate of *M. oryzae*, MG-79, and the same isolate transformed with candidate *AvrPi54* gene (transgenic isolate MG-79_*AvrPi54*) using fungal transformation vector pCB1532_*AvrPi54* (**Figure 4A**). Hence, the transgenic and the non-transgenic fungal isolate had the same genetic background except that the transgenic isolate contained the candidate *AvrPi54* gene which the non-transgenic isolate did not. Transgenic fungal isolate could be distinguished from the non-transgenic by its ability to withstand sulfonylurea selection (**Figure 4B**), but otherwise there were no morphological difference in terms of hyphal structure or the structure and number conidia produced by the transgenic and non-transgenic isolates (**Figure 4C**). At molecular level, the transgenic isolate can be identified by the appearance of 2376 bp amplicon in the polymerase chain reaction (PCR) using candidate *AvrPi54* expression cassette-specific primer-pair (**Figure 4D**). Expression of candidate *AvrPi54* transgene in MG-79_*AvrPi54* was assayed by semiquantitative reverse transcriptase PCR (RT-PCR) taking housekeeping gene, *Actin*, of *M. oryzae* as internal control (175 bp amplicon). cDNA derived from the mycelia of MG-79 and MG-79_*AvrPi54* were used as templates for RT-PCR. Appearance of 102 bp candidate *AvrPi54* gene specific amplicon in case of transgenic isolate and absence of the same in non-transgenic one confirms constitutive expression of candidate *AvrPi54* transgene in MG-79 background (**Figure 4E**). For the phenotyping studies, we used HR12 as universal susceptible genotype (susceptible control) and Tetep as universal resistant genotype (resistant control). The phenotyping experiment was repeated thrice with three independent fungal transformant for authenticity.

Expectedly, both transgenic (MG-79_*AvrPi54*) and non-trangenic (MG-79) fungal isolates were found to be virulent on HR-12 (having disease score 4–5) while avirulent on Tetep (having disease score 0–1), in all the cases. Rice line TP-309, which did not contain resistant *Pi54* allele, was found to be highly susceptible to transgenic MG-79_*AvrPi54* isolate as well as native untransformed MG-79 strain of *M. oryzae*. On the other hand, transgenic rice line TP-2, which contains *Pi54* gene in TP-309 background, showed disease symptoms only when challenged with native MG-79 isolate but not with transgenic MG-79_*AvrPi54* isolate of *M. oryzae* (**Figure 4F**, **Supplementary Table S8**). In other words, the presence of *Pi54* gene in the host and candidate *AvrPi54* gene in the pathogen produced resistance response in the host, while in all the other combinations disease developed. The phenotypic observations were further confirmed by isolating the fungus *M. oryzae* from the developed disease-lesions (except in case of TP-2 vs. MG-79_*AvrPi54* interaction as no disease lesions were produced) and carrying out PCR using the genomic DNA isolated from this *M. oryzae* isolate as template and *AvrPi54* expression cassette-specific primer-pair. The appearance of 2376 bp amplicon in *M. oryzae* derived from TP-309 verses MG-79_*AvrPi54* interaction, in contrast to the absence of that particular amplicon in case of *M. oryzae* isolated from TP-2 verses MG-79 and TP-309 verses MG-79 interactions confirmed the absence of any source of contamination in this experiment (**Figure 4F**). Our results demonstrated the complementation of avirulence function in otherwise virulent MG-79 isolate of *M. oryzae* transformed with the candidate *AvrPi54* gene in presence of *Pi54* gene in the host.

## Discussion

### Whole Genome Resequencing of *M. oryzae* Isolate RML-29

The present study was conducted with the basic objective of identification and cloning of *AvrPi54* gene from rice blast pathogen *M. oryzae* by decoding the genome of an avirulent strain (RML-29) in which we predicted 11440 protein coding genes. The number of predicted protein coding genes in the RML-29 genome was found to be slightly higher than the number of protein coding genes (11109) predicted in whole genome sequence of *M. oryzae* isolate 70-15 genome (Dean et al., 2005). This might be because we set the gene length threshold at ≥50 amino acids as we wanted to obtain as much candidates as possible for further downstream analysis. Similar approach has been followed by Yoshida et al. (2009). However, in the whole genome analysis of *M. oryzae* isolate 70-15, the length threshold was set at ≥100 amino acids (Dean et al., 2005).

### *In silico* Secretome Analysis to Identify Candidate AvrPi54 Protein

During biotrophic invasion, a range of low molecular weight biotrophy-associated secreted (Bas) proteins are secreted by *M. oryzae*, which also include the effectors. These are initially accumulated in Biotrophic Interfacial Complex (BIC) and later the cytoplasmic effectors enter the host cell in due course of time when the pathogen penetrates the host (Kankanala et al., 2007; Khang et al., 2010; Giraldo et al., 2013). BICs are hypothesized as the site of effector translocation based on the strong correlation between preferential accumulation of known effectors such as AvrPi-ta, Pwl2, and AvrPiz-t, and their delivery into the host cytoplasm (Khang et al., 2010; Valent and Khang, 2010; Park et al., 2012; Giraldo et al., 2013). We hypothesize that the AvrPi54 protein, similar to many other fungal cytoplasmic effector proteins, is a part of its secretome (Ridout et al., 2006; Doehlemann et al., 2009; Lievens et al., 2009; Stergiopoulos and de Wit, 2009; Tan et al., 2010; Valent and Khang, 2010; Kloppholz et al., 2011; Marshall et al., 2011; Brown et al., 2012; Park et al., 2012) that enter the host cell via the same pathway. Hence, we decided to analyze the secretome of *M. oryzae* strain RML-29. Body of evidences suggests that cytoplasmic effector secretion occurs through an exocyst-aided pathway, which is distinct from conventional endoplasmic reticulum (ER)–Golgi bodies (GB) secretion pathway because it involves ER but bypasses the GB (Giraldo et al., 2013). These effector proteins contain classical SP at their N-terminal, which facilitates their delivery to the ER (Mosquera et al., 2009; Valent and Khang, 2010; Saitoh et al., 2012). The *MgLHS1* gene knocked out strains, which are incapable of encoding ER chaperons, show severe impairment in effector secretion and are also incapable of inducing *AvrPi-ta-*mediated hypersensitive response (HR; Yi et al., 2009). Hence, we systematically analyzed the presence of N-terminal SPs *in silico* in the whole RML-29 proteome to determine proteins having such typical signature elements. With this analysis, we identified 735 unannotated proteins that constituted the working secretome for this study. We also employed a size exclusion screen of 330 amino acids to minimize our search space and retained only secreted proteins which are small in size (smaller than the counterpart resistance gene Pi54). It is reported that the cytoplasmic effector proteins are of small size (low molecular weight; Valent and Khang, 2010; Giraldo et al., 2013). Though the cloned Avr proteins of *M. oryzae* show a great extent of size variation (Sharma et al., 2012), but in general, the avirulence proteins are smaller in size than its counterpart resistance proteins. Barring ACE1, which is not a secretory protein but a pathogen-localized enzyme (Böhnert et al., 2004), this fact is also evident from the sizes of the cloned *Avr* gene-encoded proteins of *M. oryzae* (Orbach et al., 2000; Zhou et al., 2006; Li et al., 2009; Gururani et al., 2012; Sharma et al., 2012). Hence, we decided to fix the upper size limit to 330 amino acids, which is the size of its counterpart R-protein, Pi54.

### Employing *Neurospora crassa* Screen to Identify Pathogenicity-Related Proteins

Both conventional and the exocyst-aided secretion initiates from ER (Giraldo et al., 2013). Therefore, all the proteins destined for secretion contain N-terminal SP, which facilitates their delivery to ER. Hence, our working secretome, besides being rich in pathogenicity-related effector proteins, was also expected to contain proteins not related to pathogenicity. To remove those unrelated proteins from the search space, we designed and employed a screen, which we termed as *Neurospora crassa* screen. *N. crassa* is one of the closest non-pathogenic relatives of *M. oryzae* (Dean et al., 2005; Kasuga et al., 2009). Both these fungi belong to the class *Pyrenomycetes* (Dean et al., 2005), which is otherwise known as *Sordariomycetes* (Kasuga et al., 2009). Despite 200 million years of divergent evolution (Hedges, 2002), comparative genomic study revealed that out of 11109 protein coding genes reported in the whole genome sequence of *M. oryzae* isolate 70-15, 10082 genes were homologous to *N. crassa* with an average identity of 47% at amino acid level (Dean et al., 2005). Moreover, these two fungal genomes shared approximately 100 kb of syntenic region (Hamer et al., 2001). Another reason of selecting *N. crassa* over other non-pathogenic relatives such as *Aspergillus nidulans*, *Chaetomium globosum*, and so on, was because *N. crassa* has a mechanism, called repeat-induced point-mutation (RIP), to limit the spread of paralogous gene duplication (Galagan et al., 2003). Hence, the observed homology is expected to be a resultant of orthologous relationship only. Comparative analysis of these two genomes also reveals that *M. oryzae* has experienced an ancient expansion of gene family and acquired genes to suit its pathogenic life style (Idnurm and Howlett, 2001; Dean et al., 2005). Capitalizing on these facts, we identified and removed the common proteins (homologs) of *M. oryzae* and *N. crassa* from our predicted secretome and our search space was reduced to 370 proteins. These proteins were secretory in nature, unique to *M. oryzae* and are expected to be related with pathogenicity.

### Identification of Candidate AvrPi54 Protein by Candidate Elimination Approach

Once the pathogenicity-inducing proteins were identified, the next challenge was to identify the candidate AvrPi54 protein(s) present in this set of 370 genes. As the known *Avr* genes encodes proteins that are structurally and functionally very much diverse from each other, it is relatively difficult to identify Avr proteins in fungal pathogens. Therefore, unlike the resistance proteins, the avirulence proteins do not have any structural homology by which they can be identified. For example, in case of *M. oryzae*, out of 11 cloned *Avr* genes, protein information is available for seven. *PWL* series of Avr genes (*PWL1*, *PWL2*, *PWL3*, and *PWL4*) encodes glycine rich small secreted proteins (Kang et al., 1995; Sweigard et al., 1995), whereas *AvrPi-ta* encodes a secreted metalloprotease (Orbach et al., 2000). *ACE1* gene encoded avirulence protein on the other hand is completely different from the other proteins as it encodes an NRPS/PKS enzyme that is non-secretory in nature. It is involved in the production of toxic metabolites that function as effector molecules (Böhnert et al., 2004). Apart from these, recently it has been reported that *AvrPiz-t* encodes a cytoplasmic effector protein that interacts with APIP6 (an E3-ubiquitin ligase) and suppresses PAMP (pathogen-associated molecular patterns)-triggered immunity (PTI) by interfering with proteosomal degradation pathway (Li et al., 2009; Park et al., 2012). Although the lack of structural homology is a major limitation for the identification of Avr proteins, here we exploited this phenomenon to our advantage to eliminate the already known Avr proteins of *M. oryzae* and their close homologs from the present set of 370 effector proteins; 54 hypothetical proteins remained as probable candidates. As the *Avr* genes of *M. oryzae* are expressed in the infectious structure, appressoria (Fudal et al., 2007; Stergiopoulos and de Wit, 2009), we computational analysis of *in vivo* expression of these 54 proteins in *M. oryzae*. Among these, four proteins were found to be expressed in the appressoria of *M. oryzae* spores. These four proteins were used for generating 3D models. Amino acid sequences of mature proteins (protein after the truncation of N-terminal SP) only were used for model generation as the N-terminal SP is naturally cleaved during their entry to ER (Rafiqi et al., 2000; Rehm et al., 2001). By protein modeling of these four candidate Avr proteins and docking of these models on the model of resistance protein Pi54, we found that only Mo-01947_9 protein showed strong interaction potential at the LRR domain of the Pi54 protein. Mo-01947_9 protein model was selected as the only candidate capable of interaction with Pi54 protein model because, unlike other three candidates (Mo-02191_8, Mo-01702_7, and Mo-1127_86), its docked complex with Pi54 protein model showed negative value (-139.17 kcal/mol) of Atomic Contact Energy (ACE). ACE is defined as the sum of free energy (Δ*G*) of replacing atom–water contacts by atom–atom contacts for all the atoms taking part in the protein–protein interaction (Dodds et al., 2006). Hence, negative value of ACE suggests thermodynamic feasibility of interaction between two proteins by excluding the water hull surrounding their area of contact. We also calculated the binding energy of the complex which is defined as the free energy released during complex formation and is calculated by using the formula: Binding energy = Energy (complex) – [Energy (receptor) + Energy (ligand)]. Larger negative value suggests stronger interaction (Zhang et al., 1997). Since Mo-01947_9 protein model was the only candidate capable of interaction with Pi54 protein model (forming complex), binding energy could be calculated for Pi54:Mo-01947_9 complex only. As expected it showed large negative value (-313.54 kcal/mol). Binding energy could not be calculated for rest of the interactions because of inability of forming complexes with Pi54 protein model. Apart from docking results, the *Avr* protein Mo-01947_9 protein also corresponds to all the criteria to be qualified as a cytoplasmic effector as it is small in size (153 amino acids), has low molecular weight (15.786 kD), and is of secretory nature (Mosquera et al., 2009; Valent and Khang, 2010; Saitoh et al., 2012; Giraldo et al., 2013). Its secondary structure is composed of six β-sheets and no α-helix (mature protein), which is very much similar to linseed rust avirulent protein AvrL567. AvrL567 is a monomeric, single domain protein, which is mostly composed of β-sheets. It also contains the surface-exposed residues, which are expected to play an important role in the recognition by LRR domains of corresponding R proteins L5, L6, and L-7 (Kollman et al., 2000). Similar recognition event can be expected in case of Pi54 and Mo-01947_9 interaction too.

To check the possibility of this physical interaction (between Pi54 protein model and Mo-1947_9 protein model) leading to avirulence function, we docked the Mo-01947_9 protein model on the protein model deduced from the susceptible allele of *Pi54* gene (Pi54_TP-309). The susceptible allele Pi54_TP-309 encodes a 276 amino acid long protein. It is actually a truncated version of the protein encoded by resistant Pi54 allele of Tetep (Pi54_Tetep). As mentioned earlier Pi54_Tetep is of 330 amino acids. The major truncation in Pi54_TP-309 (as compared to Pi54_Tetep) is at the N-terminal region keeping the C-terminal region almost intact (**Supplementary Figure S4A**). C-terminal region contains the LRR domain, the key player for interaction with Avr counterpart. No interaction was found between Mo-01947_9 and Pi54_TP-309 (**Supplementary Figure S4B**). This result is also consistent with Flor’s hypothesis (Flor, 1971) making Mo-1947_9 as a promising candidate to be qualified as AvrPi54. Further, the absence of interaction even in the presence of undisturbed LRR domain certainly speaks of the involvement of other domains too (apart from LRR) in the R–Avr interaction. Therefore, it can be assumed that although the LRR domain of the R-protein is the key player in interaction between R- and Avr- proteins, the overall 3D geometry of the R-protein is also equally important to make the interactions possible for these proteins. The cooperative role of N-terminal TIR and ARC domain along with C-terminal LRR domain of resistance proteins has been elucidated in binding of AvrL567 protein (Ravensdale et al., 2012).

### Physical Interaction Helps in Recognition of Candidate AvrPi54 Protein by Pi54 Protein

We identified candidate AvrPi54 protein (Mo-01947_9) *in silico* based on its physical interaction potential with Pi54 protein. To prove this interaction further, we performed Y2H analysis. The results of Y2H clearly demonstrated the physical interaction between Pi54 and candidate AvrPi54 proteins. Our results are consistent with the earlier observations of direct physical interaction between Pi-ta:AvrPi-ta proteins in case of rice–rice blast pathosystem (Jia et al., 2000) and L6:AvrL567 interaction (Wang et al., 2007) in case of flax–flax rust. The protein products of *Pi54* and candidate *AvrPi54* genes were found to interact physically *in silico* by docking analysis and in yeast by Y2H analysis.

After detecting direct physical interaction between Pi54 and candidate AvrPi54, proteins, the next obvious question was if this interaction is capable of inducing HR following typical gene-for-gene interaction (Flor, 1971; Dangl and Jones, 2001). In rice–*M. oryzae* pathosystem evidences for direct physical interaction between R- and Avr- proteins has been demonstrated at least for two R–Avr pairs, viz., Pi-ta:AvrPi-ta (Orbach et al., 2000) and Piz-t:AvrPiz-t (Li et al., 2009). Besides, resistance gene analog, RGA5 (the suppressor of RGA4 mediated hypersensitive cell death), is also reported to induce resistance response during rice–*M. oryzae* interaction by directly interacting with two different avirulence proteins, AvrPia and Avr1-Co39 (Cesari et al., 2013). Agroinfiltration has long been used as a versatile system to study R–Avr interaction and *N. benthamiana* has been used as a preferred host for several advantages (Van der Hoorn et al., 2000; Burch-Smith et al., 2007; Ravensdale et al., 2012). We performed a leaf infiltration assay where *Pi54* gene and candidate *AvrPi54* gene encoding Mo-1947_9 protein were transiently co-expressed via agroinfiltration in the leaves of *N. benthamiana*. Co-expression of *Pi54*- and candidate *AvrPi54*- genes induced strong HR in tobacco but HR was not detected when these two genes were expressed individually. Hence, the obtained results clearly suggest that physical interaction between Pi54 protein and Mo-1947_9 (candidate AvrPi54) protein is capable of inducing HR.

Finally, to be qualified as a candidate *AvrPi54* gene its avirulence function has to be demonstrated in *M. oryzae*. Two approaches are generally used for this purpose. Firstly, by using knock-out mutants, for example, in case of *ACE-1* (Böhnert et al., 2004) a deletion mutant (ace1Δ:hph) was constructed, and *M. oryzae* strain was transformed with it. The transformed strain was unable to induce avirulence response against cognate resistance gene *Pi33*. The second approach is by employing genetic complementation test, which is more commonly practiced and was used in case of *Avr1-Co-39* (Farman and Leong, 1998), *AvrPiz-t*, and *AvrPia, AvrPii*, and *AvrPik/km/kp* (Yoshida et al., 2009). As a general strategy of genetic complementation test, a virulent strain of *M. oryzae* is transformed with the candidate *Avr* gene and the avirulence function of the candidate gene is considered to be validated, if the isolate is unable to infect rice plants containing its cognate resistance gene. In this case, the candidate gene is able to complement avirulence function in the otherwise virulent isolate. We used the later approach, that is, the complementation test to validate the avirulence function of candidate *AvrPi54* gene in our study.

TP-2 is a transgenic rice line, which contain functional *Pi54* gene in Taipai-309 (TP-309) background (Rai et al., 2011), hence, is resistant to *AvrPi54* containing *M. oryzae* isolates. *M. oryzae* isolate MG-79 does not contain active *AvrPi54* gene in its background, but when it was transformed with candidate *AvrPi54* gene (encoding *Mo-1947_9* protein) cloned from avirulent isolate RML-29, the transgenic isolate MG-79_*AvrPi54* became avirulent on TP-2 lines containing dominant blast resistance gene *Pi54*. Our results of complementation test are very much consistent with the earlier mentioned complementation studies. In one of such studies, Cosmid 1803 containing candidate *Avr1-Co39* was used to transform Guy11 isolate, which is virulent on *Co39* containing rice lines. The transformed isolate turned avirulent on the same *Co39* containing rice lines, which confirmed the avirulence function of *Avr1-Co39* (Farman and Leong, 1998). Similarly, transformation of Guy11 isolate with 7bg7.15 (candidate *AvrPiz-t* gene) converted it to an avirulent form on *Piz-t* gene containing transgenic Nipponbare rice lines, confirming the avirulence function of *AvrPiz-t* (Li et al., 2009). The function of other cloned *Avr* genes was also confirmed based on their ability to complement avirulence function in virulent isolates of *M. oryzae* in the presence of corresponding resistance gene (Yoshida et al., 2009). Our results confirm that the candidate AvrPi54 protein (Mo-01947_9) not only physically interact with the Pi54 protein and induces HR but also complements avirulence function *in vivo*, in presence of cognate resistance protein. The present study thus indicates that the candidate *AvrPi54* gene (encoding Mo-01947_9 protein) identified and cloned from *M. oryzae* isolate RML-29 fits all the necessary criteria to qualify as true *AvrPi54* gene and it plays a role in triggering host resistance probably by directly interacting with resistance protein, Pi54. Results of yeast-two-hybrid analysis and *N. benthamiana* leaf infiltration assay indicated that Pi54 protein and Mo-01947_9 protein directly interact with each other and induce hypersensitive cell death in host. Hence, it is a typical example of gene-for-gene system (Flor, 1971). We used genome-wide *in silico* analysis, protein modeling, and *in silico* docking to identify the candidate *AvrPi54* gene. To the best of our knowledge this approach has not been previously used for the identification of avirulence gene from any plant pathogen. The approach adopted in the present study has a great potential to reduce the time and labor involved in identifying and mapping an *Avr* gene especially in model systems where genomic resources are available and could be used as an alternative to the more tedious positional cloning approach. The candidate *AvrPi54* gene identified and cloned in the present investigation would be helpful in better understanding of the host–pathogen interaction. As the *Pi54* gene is already cloned and functionally validated, the newly identified *AvrPi54* gene, along with *Pi54* gene, could be used in constructing *R–Avr* two components system (Sharma et al., 2012) to develop broad spectrum resistance against rice blast pathogen.

## Materials and Methods

### Plant Materials and Fungal Material

The susceptible rice cultivar Taipei-309 (TP-309) and transgenic rice line TP-2 (containing *Pi54* gene in TP-309 background), were used in this study. These were available in our laboratory and were grown and maintained under controlled conditions at the National Phytotron Facility, New Delhi, India.

*M. oryzae* strains MG-79 (Strain ID: Mo-ei-79; kindly provided by Dr. M. Variar) which is virulent and RML29 (Strain ID: Mo-nwi-55) which is avirulent, on host plant containing blast resistance gene *Pi54* were used in this study. The fungal strains and isolates used in this study were maintained by culturing in Petri plates containing oat meal agar (60 g/L of oat meal and 12.5 g/L of agar) medium and incubated at 25°C in dark. Subculturing was performed on same medium at 21 days intervals.

### DNA Isolation

For DNA isolation, fungus was cultured in liquid medium containing 3 g/L of yeast extract, 3 g/L of casamino acid, and 10 g/L of glucose. The cultures were maintained in conical flasks (250 ml) in dark at 37°C for 7 days on a rotary shaker (100 rpm).

Before transformation of fungus, the fungal mycelia were grown in liquid complete medium for 5 days (Talbot et al., 1993). After transformation, TB3 media (0.3% Yeast extract, 0.3% Casamino acid, 1.0% Glucose, 20% Sucrose, and 8% agar) was used for protoplast regeneration under dark conditions. For subsequent subculturing, complete medium (liquid complete media with 15 g/L agar) was used.

For inducing sporulation in the transgenic as well as non-transgenic cultures, Mathur’s medium (glucose 2.80 g/L; MgSO_4_, 7H_2_O 1.23 g/L, KH_2_PO_4_ 2.72 g/L, Peptone 2.00 g/L, and Agar 20.00 g/L) was used. The plates were kept under light after culturing for 10–15 days to induce sporulation.

Five-day-old fungal mycelia (∼1 g) were harvested and ground well to fine powder in liquid nitrogen. Genomic DNA was isolated from the ground mycelia of *M. oryzae* by *N*-Cetyl-*N*, *N*, *N*-Trimethyl ammonium bromide (CTAB) method (Rogers and Bendish, 1988).

### Genome Sequencing and Assembly of *M. oryzae* Isolate RML-29

The genome RML-29 was sequenced using 454 GS FLX sequencing technology (13-fold). Filtered high quality sequence reads were assembled through “GS Reference Mapper” version 2.3 (Roeche Inc. Germany). In assembly, the minimum overlap match length, minimum overlap identity, and total number of Central Processing Unit (CPU) utilized for this purpose were fixed as 40 bp, 90%, and 0 (all). The supercontig 6.1 sequences of *M. oryzae* isolate 70-15 genome^5^ were considered as reference in this assembly of RML-29 genome. MUMmer4.0 software (Kurtz et al., 2004) was used to align total assembled RML-29 contigs on the build3.1 of reference genome. We filtered the chromosome-specific mapped contigs and created chromosome-wise pseudo scaffolds by passing them to a set of perl scripts and also identified RML-29-specific unique contigs that did not match with the reference genome. All the unique contigs sequences were taken as query and were further subjected to the database of raw reads of the reference genome^6^ in the BLASTN (N-nucleotide) program of ncbi-blast-2.2.28+ package for seeking their extent of uniqueness present in the RML-29 genome^7^.

### *In silico* Identification of *Avr* Genes in *M. oryzae* Genome

We generated high quality (≥Phred 20) whole genome sequence of an avirulent strain of *M. oryzae* using Pyrosequencing (454 Life Sciences, Roche Applied Science, Basel, Switzerland; Margulies et al., 2005). The supercontigs obtained after assembling the sequence reads were used for gene prediction using FGENESH^8^ taking *Magnaporthe* as reference database for gene prediction. Among all the predicted proteins, the proteins capable of sorting were identified using PrediSi software^9^. Size exclusion screen (to select the sorted proteins within the size range of 50–330 amino acids) was performed in MS excel. Secretory proteins were filtered out from the sorted proteins using TargetP^10^. Unannotated secretory proteins were identified by BLASTP^7^ against non-redundant protein database using the secretory proteins as queries and removing the proteins from the list having high scoring hits (bit score > 100, *E*-value > *e*^-20^, and similarity > 30%) in the database with already annotated proteins. To perform *N. crassa* screen, the proteome of *N. crassa* was downloaded from NCBI database^7^. BLASTP^7^ was performed to identify the proteins common in *N. crassa* and *M. oryzae* using the unannotated secretory proteins as query and *N. crassa* proteome as subject. Hits having bit score > 100, *E*-value > *e*^-20^, and similarity > 30%, were considered as common proteins and were removed from the list. After this analysis we got unannotated secretory proteins unique to *M. oryzae*. Finally, to find secretory proteins unique to *M. oryzae* that are expressed in appressorium, BLASTN^7^ was performed against EST database (est_others) using the CDS of the identified unique proteins as query. The CDS showing significant match (bit score > 100, *E*-value > *e*^-20^, similarity > 70%, and query coverage > 50%) to the ESTs solely expressed in the *M. oryzae* appressorium were considered as originating from the genes solely expressed in appressorium and were used for further protein modeling and docking studies.

### Protein Modeling of Selected *AvrPi54* Candidates and Docking against Pi54 Protein

The structures of selected proteins were developed through homology-based protein modeling by using Accelrys Discovery Studio 2.1 software^11^. To identify the homologs of the candidate proteins, HHPred software^12^ was used. The Protein Data Bank (PDB) database^13^ was explored to determine and download the structural homologs of the proteins to be modeled, which served as template files. The amino acid sequences of these template proteins were also downloaded and stored in a file along with the amino acid sequence of the query sequence, for which 3D protein structure has to be modeled. Multiple sequence alignment (MSA) of these sequences was performed to create an alignment file. This MSA was performed by ClustalW inbuilt in Accelrys Discovery Studio 2.1 software. The matrix used for alignment was BLOcks SUbstitution Matrix (BLOSUM) 30 with gap opening penalty 10.0 and gap extension penalty 1.0.

The template file and the alignment file were uploaded to the MODELER program, which is again inbuilt in Accelrys Discovery Studio 2.1. Specific links were provided to link each of the template sequence in the alignment file to its corresponding structure in the template file. Using the linked template file and alignment file as the inputs, MODELER generates the 3D tertiary structure of the query protein as an output with all details. An energy minimization step was carried out to refine the obtained structure and bring it down to lowest energy state, that is, the stable conformation. This energy minimization was performed through CHARM27 force-field, which is again inbuilt in Accelrys Discovery Studio 2.1.

Docking analysis was carried out through Z-Dock program, inbuilt in Accelrys Discovery Studio 2.1 software. For this purpose, the 3D models of Pi54 protein and its candidate Avr counterparts were uploaded as the inputs.

### Yeast-2-Hybrid Analysis

*Pi54* CDS and candidate *AvrPi54* CDS were amplified using specifically designed primers carrying desired restriction sites in it (**Supplementary Table S9**) to facilitate cloning in Y2H vectors, pGBKT7, and pGADT7 using manufacturer’s instructions (Clontech, USA). Purified PCR products were cloned in pGBKT7 and pGADT7 vectors using standard protocol and transformed in *E. coli* strain DH5α cells prepared by using Z-Competent^TM^ *E. coli* Kit (Zymo Research Corporation, USA). The positive transformants (containing the desired insert) were selected through colony PCR and restriction analysis. The plasmid DNA extraction was performed by High Speed Plasmid Mini kit following manufacturer’s protocol (Geneaid Biotech Ltd, Taiwan). Y2H analysis was performed using Clontech Matchmaker^TM^ gold Y2H kit (Clontech, USA) following manufacturer’s protocol.

### Tobacco Leaf Infiltration Assay

Candidate *Avr-Pi54* gene expression cassette was prepared by placing SP truncated candidate *Avr-Pi54* coding sequence in between 35S promoter of *Cauliflower mosaic virus* (CaMV35S) and nopaline synthase (NOS) terminator and the cassette was cloned in MCS of the binary vector pCAMBIA1305.1. Similarly, *Pi54* gene expression cassette was prepared by placing *Pi54* coding sequences in between the CaMV35S promoter and NOS terminator and was coned in the MCS of pCAMBIA1305.1. Proper vector construction was confirmed by restriction digestion as well as sequencing. Both of these vectors (containing candidate *AvrPi54*-CDS and *Pi54*-CDS) were mobilized into *Agrobacterium tumefaciens* strain GV3101, individually. Vector mobilization in *A. tumefaciens* was confirmed by colony PCR using gene specific primer-pairs. Starter cultures from the confirmed colonies were kept at 28°C with shaking at 200 rpm for 14 h in LB medium. Cultures suspension for agroinfiltration were prepared by harvesting *A. tumefaciens* cells at OD_600_ reading of 0.8 and resuspending the harvested cells in resuspension buffer containing liquid MS medium and 100 μM acetosyringone. Finally, agroinfiltration was performed by slowly infiltrating the prepared *Agrobacterium* suspension into 4 weeks old *Nicotiana benthamiana* leaves. Tobacco leaves were infiltrated with *A. tumefaciens* cultures containing candidate *AvrPi54* and *AvrPi54* gene expression cassettes, separately as well as simultaneously. For co-infiltration, equal volumes of the two *A. tumefaciens* cultures carrying the candidate *Avr-Pi54* and *Pi54* genes expression cassette were mixed together before infiltration into *N. benthamiana* leaves. To nullify any effect produced by the binary vector (pCAMBIA1305.1) in this assay, an empty vector was also infiltrated independently in tobacco leaves as control. Results were recorded after 4 days post infiltration.

### Construction Of Expression Cassette For Fungal Transformation

The fungal expression cassette was designed by fusing full length candidate *AvrPi54* gene (1310 bp) with CaMV35S promoter (870 bp) upstream and NOS terminator (289 bp) downstream using overlap-extension PCR (OE-PCR). Primers were designed in such a way that *XbaI*_CaMV35S_R contains overlap with *XbaI*_AvrPi54_F and *XmaI*_AvrPi54_R contains overlap with *XmaI*_NOS_F to facilitate joining of the fragments. The restriction sites *XbaI* and *XmaI* were provided with the primer to check the authenticity of joining of the amplicons after each round of OE-PCR, whereas *BamHI* and *EcoRI* sites were provided to facilitate cloning of expression cassette in pCB1532 vector specialized for fungal transformation (kindly provided by Dr. N. J. Talbot). The details of primers used in this experiment are given in **Supplementary Table S9** (restriction sites are marked in bold).

In the first round of OE-PCR, CaMV35S amplicon (*BamHI*-CaMV35S-*XbaI*, 870 bp), candidate *AvrPi54* amplicon (*XbaI*-AvrPi54-*XmaI*, 1310 bp), and NOS amplicon (*XmaI*-NOS-*EcoRI*, 289bp) were amplified separately (**Supplementary Figure S5**). For CaMV35S and NOS, plasmid vector pBI121 (which contains CaMV35S promoter and NOS terminator in its backbone) was used as template, whereas for candidate *AvrPi54*, genomic DNA of *M. oryzae* isolate RML-29 was used as template for PCR amplification. These amplicons were eluted and purified from agarose gel for use in second round of amplification. In the second round of OE-PCR, CaMV35S and candidate *AvrPi54* were joined by using *BamHI*-CaMV35S-*XbaI* and *XbaI*-AvrPi54-*XmaI* as template and *BamHI*_CaMV35S_F and *XmaI*_AvrPi54_R as primer to obtain *BamHI*-CaMV35S-*XbaI*- AvrPi54-*XmaI* amplicon of 2134 bp (**Supplementary Figure S5**). The authenticity of joins was confirmed by digesting the fragment with *XbaI*, which released *BamHI*-CaMV35S-*XbaI* (870 bp) and *XbaI*-AvrPi54-*XmaI* (1310 bp) fragments after digestion (**Supplementary Figure S5**). In the same manner, candidate *AvrPi54* and NOS were joined by using *XbaI*-AvrPi54-*XmaI* and *XmaI*-NOS-*EcoRI* as template and *XbaI*_AvrPi54_F and *EcoRI*_NOS_R as primer to obtain *XbaI*-AvrPi54-*XmaI*-NOS-*EcoRI* amplicon of 1552 bp (**Supplementary Figure S5**). The authenticity of joined fragments was confirmed by restriction digestion using *XmaI*, which released *XbaI*-AvrPi54-*XmaI* (1310 bp) and *XmaI*-NOS-*EcoRI* (289 bp) fragments (**Supplementary Figure S5**). Finally, in the third round of OE-PCR, *BamHI*-CaMV35S-*XbaI*-AvrPi54-*XmaI* and *XbaI*-AvrPi54-*XmaI*-NOS-*EcoRI* were joined together to obtain a complete expression cassette by using these two as templates and *BamHI*_CaMV35S_F and *EcoRI*_NOS_R as primers. This PCR reaction yielded a complete expression cassette (*BamHI*-CaMV35S-*XbaI*- AvrPi54-*XmaI*-NOS-*EcoRI*) of 2376 bp (**Supplementary Figure S5**). Proper construction of the expression cassette was confirmed by double digestion with *XbaI* and *XmaI*, which released *BamHI*-CaMV35S-*XbaI* (870 bp), *XbaI*-AvrPi54-*XmaI* (1310 bp), and *XmaI*-NOS-*EcoRI* (289 bp) fragments (**Supplementary Figure S5**). About 100 ng template DNA was used for all the PCR reactions. Each PCR reaction consisted of 2.5 μL each of forward and reverse primers (10 μM each), 10 μl of 5X phusion buffer HF, 1 μL of 50 mM MgCl_2_ (Finnzymes, Finland), 1 μL of 10 mM dNTP mix (MBI Fermentas, Lithuania), and 1 U of Phusion DNA polymerase (Finnzymes, Finland). The volume of reaction mixture was made up to 50 μl by adding sterile water. The thermal profile of the PCR reactions was set according to manufacturer’s protocol with standardized annealing temperature for each primer pair. The full length expression cassette was cloned in pCB1532 vector at *BamHI-EcoRI* site to produce pCB1532_*AvrPi54* using standard procedure of cloning and transformed in *E. coli* strain DH5α cells prepared by Z-Competent *E. coli* Kit (Zymo Research Corporation, USA). The positive transformants (containing the desired insert) were selected through blue/white screening and restriction analysis. The plasmid DNA extraction was performed by High Speed Plasmid Mini kit following manufacturer’s protocol (Geneaid Biotech Ltd, Taiwan).

### Transformation of Virulent Isolate *M. oryzae* with Candidate *Avr* Gene

Protoplast transformation method (Burbano-Figueroa, 2011) was followed for this purpose with necessary modifications. In brief, fungal competent cells were prepared from 5-day-old mycelia that were grown in 50-ml liquid complete medium (Talbot et al., 1993) containing chloramphenicol (200 μg/ml) at 25°C in dark conditions. Mycelia were harvested using Miracloth and washed by washing solution. Protoplast was obtained by incubating 1g of mycelia re-suspended in protoplasting solution for 24 h (30°C with gentle shaking) which containing lysing enzymes from *Trichoderma harzianum* (L1412, Sigma, St. Louis, MO, USA). Protoplasts were harvested as palettes by performing cold centrifugation (4°C) at 3000 rpm for 30 min and re-suspended in 1X STC buffer which served as competent cells of *M. oryzae*. Transformation of the isolated protoplast was performed by taking 5 μg of plasmid DNA that was mixed with two volumes of 2X STC and added in 15 mL cap tube containing 200 μL of ice-thawed protoplasts (5 × 10^7^cells/mL). This solution was gently shaken (120 rpm) and incubated at room temperature (24°C) for 10 min. After this, 1 mL PTC (2X STC plus 2X PEG) was gently added in the tubes. This solution was mixed and incubated at room temperature for 20 min. After the incubation was over, 3 mL of liquid TB3 media was added and incubated at room temperature by gentle shaking for 6 h. Regenerated protoplasts were mixed with TB3 molten agar (agar concentration 8%, temperature 40°C) containing 250 μg/mL of sulfonyl urea and plated. After 1 week of incubation at 22°C in dark conditions, filamentous colonies were transferred on complete media plates supplemented with sulfonyl urea at the rate of 250 μg/ml. All the media and solutions used for *M. oryzae* transformation is mentioned in **Supplementary Table S10**.

### Semiquantitative RT-PCR

cDNA was synthesized using 2 μg of total RNA extracted using the Trizol method (Invitrogen) from the mycelium of transgenic and non-transgenic isolates, grown for 2 weeks on oatmeal agar. As an internal control, housekeeping gene *M. oryzae* actin (MGG_03982) was amplified using MgActin_F and MgActin_R primers (**Supplementary Table S9**). Primers sqRT_AvrPi54_F and sqRT_AvrPi54_R were used to amplify the candidate *AvrPi54* gene (**Supplementary Table S9**). Fifty nanograms of cDNA were used as template in each Semiquantitative RT-PCR reaction.

### Fungal Inoculation and Phenotyping

For assaying the complementation test of candidate *AvrPi54* gene for its avirulence function, rice line –TP-309 (susceptible to *AvrPi54* containing *M. oryzae* strains), transgenic rice line TP-2 (containing *Pi54* gene in TP-309 background and resistant to *AvrPi54* containing *M. oryzae* isolates) were used in this study. The 15-day-old seedlings were inoculated by spraying suspension of conidiospores obtained from *M. oryzae* strains MG-79 (non-transgenic virulent strain) and MG-79_*AvrPi54* (transgenic strain containing candidate *AvrPi54* gene in MG-79 background), respectively. Three independent fungal transgenic cultures were used for the inoculation study. HR-12 and Tetep were used as universal susceptible and universal resistant controls, respectively. Conidial suspension of approximately 10^5^ spores/ml in 0.25% gelatine solutions were sprayed separately for each strain in such a way so that the leaves were covered with fine droplets. The experiment was carried out under controlled growth conditions at 25 ± 1°C and 90% Relative Humidity (RH) for 24 h in dark and then shifted to 16/8 h light/dark regimes. Disease reaction was recorded after 7 days of inoculation by using 0–5 disease assessment scale (Bonman et al., 1986). The inoculation experiment was repeated thrice independently to confirm the results (**Supplementary Table S8**).

## Accession Number

The whole genome sequence of *M. oryzae* isolate RML-29 can be obtained from National Centre for Biotechnology Information (NCBI) under the bioproject accession number AZSW00000000. The sequence of the *AvrPi54* gene identified and cloned in this study has been submitted to European Nucleotide Archive (ENA) of European Molecular Biology Laboratory (EMBL) Nucleotide Sequence Database under the accession number HF545677.

## Author Contributions

SR carried out the whole work and prepared the manuscript. PS generated the whole genome sequence data of *M. oryzae* isolate and performed tobacco leaf infiltration assay and phenotyping. DG, AM and CS helped in bioinformatic analysis. RR identified and provided the *M. oryzae* isolate. NS provided the scientific inputs during manuscript preparation. TS conceived and coordinated the work. SR and TS wrote the manuscript. All the authors reviewed the manuscript.

## Conflict of Interest Statement

The authors declare that the research was conducted in the absence of any commercial or financial relationships that could be construed as a potential conflict of interest.

## Footnotes

1 http://www.knowledgebank.irri.org/training/fact-sheets/pest-management/diseases/item/blast-leaf-collar

2 http://www.broadinstitute.org/annotation/genome/magnaporthe_grisea/MultiHome.html

3 http://www.predisi.de

4 http://www.cbs.dtu.dk/services/TargetP/

5 www.broad.mit.edu/annotation/genome/magnaporthe_grisea

6 ftp://ftp.ncbi.nlm.nih.gov/pub/TraceDB/magnaporthe_grisea

7 http://www.ncbi.nlm.nih.gov/

8 http://linux1.softberry.com/berry.phtml

9 http://www.predisi.de

10 http://www.cbs.dtu.dk/services/TargetP

11 www.accelrys.com

12 http://toolkit.tuebingen.mpg.de/hhpred

13 http://www.rcsb.org/pdb/home/home.do
