# Supplementary material for: Analysis of Magnaporthe oryzae Genome Reveals a Fungal Effector, Which Is Able to Induce Resistance Response in Transgenic Rice Line Containing Resistance Gene, Pi54
Source: Front Plant Sci. 2016 Aug 8;7:1140. doi: 10.3389/fpls.2016.01140 (PMC4976503; doi:10.3389/fpls.2016.01140)
Supplement: Supplementary file 1 [file Presentation_1.PDF]

Supplementary Material: Analysis of *Magnaporthe oryzae* genome reveals a fungal effector, which is able to induce resistance response in transgenic rice line containing resistance gene, Pi54

Soham Ray, Pankaj K. Singh, Deepak K. Gupta, Ajay K. Mahato, Chiranjib Sarkar, Rajeev Rathour, Nagendra K. Singh and Tilak R. Sharma

Supplementary Table S1: List of selected 54 candidate AvrPi54 proteins along with their amino acid sequences. The sequence of N-terminal signal peptide (SP) for each protein is italicised and marked in bold. The ID of proteins which were further selected for modelling and docking analysis are marked in bold.

Protein ID	Length of protein (aa)	Length of SP (aa)	Amino acid sequence
Mo-00831_7	82	22	***MKSATIFATVATLALLPFGALA***GKWRCNVKIYNDKRKYQGQASDDWGETLTIRGYTCYTDSYCKASCNGLPAGWTAEGTQLN
Mo-00952_14	184	21	***MKFNSGLLAGAAVLVAGVATA***QDCISVALSAIPSCAQPCFLNGAPTIGCSGTDFKCQCQQQAKMFAAVESCVQKSCPESEFQKTIDGSDKVCTCASGGPASNNAGGAGNTVNPSSFIPGPTSTASPTTTVAAPTGTPSGRPSAVPTAAANMAAVECSIVVGAVGGALWVALGLTPRLIISQDPT
Mo-00481_2	176	18	***MGIFQLFALGVWPATAVA***SPFFAYSPEQLAKENPPQAKLQSNSPAATTDSHHVVPDKRATDAAPQLMARGCTTTIRNNNIPCYWDGTETLYSATRTMTAPVDCHGCSDIYIRQDVYFCPVEKIYTTLWAPNPSTTWITVCAASTQKAVNLNKQDILTNDAALPGRTRAPHPVPTGI
**Mo-01702_7**	**103**	**21**	***MQFKSALALSIAALQAVPALA***MEDGCSASILKYDANGGQETYYGSALPANGSIAFQPTEFSTEVVISVDANCNPTNTDDVKKSVPKGWGLHVYKKVTAIAGES
Mo-02266_3	67	22	***MQLATVFAAAATLAVLPTGVLA***GPRYIPHPECEVTIMKPGTPSPRSPYTLPEEYDRGYGPGDSKTKI
Mo-01300_5	127	21	***MQFRSIFAIVIAATHTAPVLA***GKKGDCFMGLYEAGGRNPNHIIGQETPDDSGIFELRRENGSIIQVKVKKCQLVAYWGWVPLGQELRVFPSVGYDAKRKTAPLPSLSPNRHSGQGLKVRGANTWIAF
Mo-01702_9	82	25	***MQFSQILTVLFLGVSVSALPAGGLP***GSPGSAVQRCHCPPRGSHAHGSLAAREEAPEAEGDAKISARYTCPNCHKTGKGCDDG
Mo-00684_1	190	18	***MRFSSVFALSALIQAAVS***APIPSGTGTGNAVNAITDAATDSTPLKVATGVTATGGALYGLTGQGLASGVANGQAAVEAGEKTQAIGEEIGSRETQLAGLKDKQAGRKVATKGQTATNLNPVKGFQVWKEGGDVRRKAEGNLENAENDKAGIPRPGASGCKRSLGSACNNPALASGSSGNPSAAAAAAAKN
Mo-01361_2	77	20	***MQFSTILSLVCFGASTAVLA***AGVPAIQARGDIPFCQANHP

			SSHCTSTEACEQACGGAAQTSGCSSLCVCVCSDGRKL
Mo-01702_7	103	18	***MQFKSALALSIAALQAVP***ALAMEDGCSASILKYDANGGQETYYGSALPANGSIAFQPTEFSTEVVISVDANCNPTNTDDVKKSVPKGWGLHVYKKVTAIAGES
Mo-01702_12	114	26	***MLANNILILGLAAIASAQSFVGFAAG***GITCETGVSATQDEVVAAMVGPKGTLTEKRADNLATKGCNKVKAPLFKVSVQKKFILSYAFDKPTNTYTLCSVSIGGTGRGPQCSAKP
Mo-00541_1	147	64	***MGSATSKEMAAAVLIPLPFAVISMLLRFWIRARKNTWGPDDWAMVATVPVWLVTTISTIAMAWS***GIGQQDDTLTPEQMVNSLRWFYIFQECWCFVLVTLKWSIGFTLLRIAGGKRWVTWLIYSCLVLVTICTGGTGMFLFFGCYPVE
Mo-00288_9	113	20	***MRSSTLLIVPFYFLAGLVAA***SADKAHDIELDFEGPPSGWVCCDAGAEDADGACKAKGLNAFCCGPFKADKKRPGKGNSGCDPFFATVPTGRDVKFLNGFCTAGGDLPGHVGCA
Mo-01992_3	153	19	***MVAKGLLLALLPLVPLTAA***APTCGPNTLERRAGRGIMGIGDIRQDECGPGRSCRRPSAGPAPGRYQSGPCERIGAYLDSVGGLVPPSPSVSGQGRRLDETVVYDDQGNPRWANHNKPDPRRAAAQAAMARAKPQKASGAGSSKSKKKSFFGLF
Mo-02164_23	166	27	***MAFGLITILHIVAIVFALIELGLTAYA***VSVFYNRWGPYVVSRSPDSLNFMVFNSVWSLLVLAYLGLTPKYAARLFHKLVALGLLALTTIFWFAGSIALAAYLGGPWNCGRDTLCGSTSAAVAFGFFLWAIFTVLLVLEGLEWNRSRNSVASNPHKPHASTTPYNGA
Mo-00552_8	195	18	***MYFSKLLLLVAEITAVAA***LPSPLALEPRQDVVVVKNGSFNEVMGLGRDLRINLIYPSGANGAVMVEYQHGKDRSLLTSQNRAPAPAPAGFFPLEPVSYRMTMEGTNGGSNFDFLKIDYILNADKVGKLNIRNGIFAKFNNGQGAFSVATDGPLEYVSNSERTMLSMDLGRKGSFDGEWGFFIPTADAIAGGLTTA
Mo-01733_6	75	18	***MHFSTISILAFLATGAVA***TPVPSPNGDALQTVNEAHLLARAGVPPQENPCTGKGKVSCFFMGTTCQIQNGECILG
**Mo-02191_8**	**267**	**21**	***MVSLKLTGLAMLAFQLMGAFA***QAEGTAEPQTPAEPQLKLDVATTFPDADIFGVKLVNGRPTKAVVDLTNNEADPVRLALVTGALTNPDGLAEGAPVSDAIIRNLTAVKYDVEIPAGEKRSVTYSFALDMQPRDVVVELLAIVINAKGQTFQVPAHADKASVVEPPTSFFDPQIIFLYLVLSAAFAGTLYFVYKTWIEALFPQAKRPRAQQPKKVVKVEAAEPLSGNESAGAASGADFDAKWIPDHHINRPVAKRVKSSASAKAKKGE
Mo-02196_64	282	23	***MPPHSTAQLLLLLLATTIQPAAA***IPATPTAKTTVTTTCTTSSTTHNSLLNLGYPGYDTLTRIATTTTTIIEPPLPPQTTTGSGPIATVLETHLVAGTDTTYLVRSTTSTSTRSSAWSYTTIRTWIVHPFTAQPGSVAGPPPPPPPPQQPQPQAVAAPTPVCPETSCSNPSEEADKVRSQREAADSRCATRNMETGCQAQQCQARQAGEVEGGRDYNWWCLRISQNDFIFPELRMGRSCWSAADNSFMQLNTPCLAADYQAGCRPCRGRDLGWNGANWIDAPL

Mo-00870_1	140	19	***MRPQFIIAGVFALVANASA***MEASDAKLRPRADIDLSPMNNLMTYDQCRDSCVSGFERSGGVPRNFEVICYDKCLHLLKEQAKFATANYQAALKFPGPRMNTEKRQELGRRYLGMLFRTVKAKKHTCKVKVGPMSQKNLIV
Mo-01980_82	295	17	***MRLIALLGLGMQAMVLA***APATASKSMMYKEEEWTIRDLSRSCGHRRHDDDRHGNDHDYDHDYEHGKDREKGRGKGRGHRHGRDHGKGYDHGRNGYHDDDRDGYDEGRKSQNGHYGSNRNGYNPHKHRNTEKPSRQHRDGKDGYHDDDRRKGYTASDESHGPRAKLSLNKTLDANGGAAVANATTQLHATTTTVRHGHDDEARCRWSFTVVTQQRGRQRCGFEAGWRASAAEVRCGGHFAVAGSWSGQFGEDQGFTTLSVVDRERRLVVWPAYRDAQLEGGRVVRPDQSYVPQRLP
Mo-01809_3	218	21	***MKATSMILLSFGALAAAGSNV***AGGYGNDDTVPAPAPAVSSPAPASAVKPVAGEPSGAAKKSDDEDCETKPVPAPMATPSAAAPPSAKKVPNSDVAPAAQSSSSTPTAKPAMEKTLVISVPSVTMPAALSKPSILNDITSLIADMKPTSMIKVPGAAKPTQSAYMAAPAPAPAAPAAPAPAYNATTPKPAPVTAGAVSVATGVGAATFVAAGFMAALLF
Mo-02035_52	101	23	***MHFNTDFFAIALVALLQFGTATA***VCDFIIKQNGTQITSGNIWAGATANFLVSGKWAVVSATSECKLSLSGLPNNESYTILPTQDEETEGEDIEQADTPEAS
**Mo-01947_9**	**153**	**19**	***MQFTATIISILATAATVLA***APVEVASATPLEARACKTVAASPSGVWVSKNAPQWVGFWVPTDAVGACELKAVFPAGFPIAGTRPQVNIIDVNGPAPGSIVGTVTFTPGTSTTINSFACRQDMQYKLEVASWQAQGSVSYPAQHGTGLILTYGC
Mo-01348_1	125	21	***MHSASFLATAVIAGMASPVLA***VALPADPAPAPAPVRPVFGCFVRMSIVTTDKDSTSINSGDLIPTTNPGQVDFKDGSDTITVKLDKNCQPVGNIKHRKEVQFNALQKLTKNGGPFTDPDQPFITF
Mo-01622_43	143	18	***MISTPLLAIAIAAAVGST***QELVAAPEGYSKVYITSNVNTKFVVQPKAASVGSTLVVQQLNGNPEQQWYIKAGKTKIQLVDTTWCMDAGLKANWRNMANINLANCSDAVDGQKWNAMADGRIALELSPQPPARGWGTLEPPTRG
Mo-01492_64	146	20	***MLPQTFLLVVAAARVASTLA***IQSSQLSDLQPTPCTPSTCGHNGSVVCYNDEQYCHAEADKPNYVGESLGLPCRNDTVSPKYPITKACEESKLAPYDKDKIPSECLNTLCAAMDGKAKGLWCLYFPKGWTGLQYKKLGMECDPSEGR
Mo-01643_3	244	19	***MKSLFLTLGLLGLSASALA***EKCGDANYDPKSYICHNGNFLCPVVNGEGLSYCNGACYSKFMYQCNSGTLSQLPLLEQGSKFTLTAWNPASPSVHGKAIANSGRHWWVGGETSSYCPDIVKDQGACPPGTVTAMAYSGGMNTLVPGGQGYYLNANWNVEVTQAHSSYIPSGSTVGGLVAYKNGGFINTNGQPTGWVACPGGSDGRWNLVAGNASSADALKSCTGINLQVNYLGDSSVPASAWQYI

Mo-01956_6	188	24	***MLSSSIFVYALALVASVTPQLAHA***VPLTDGLAPRGPQSFS LPDLTKYLNPSAGNKAGSGAASSCPSTGGGDLTQKTLCS TGTSYCCVSEARGNTCIDATTKCDHTVVCCNNANGSQM CMGDVNFNMPMAINMPININIGVPSKNKDGKMRRSGIQ VLHPEGSSKGSPINSVTGLPVSDLVLDLLKTTQ
Mo-01514_2	139	18	***MHVKTTCIIAALLPISLA***APSIYSKSLATTKPHEYSNIILHH RGVPLFNIEKSESKCMGCLPKYRVRPDPGTSATFWNGVE DGQAARARFFKKAGDKCKAGFQKAADCTGLGRKAGTA KEAGAGEKARAETVGSNEGKE
Mo-02196_3	200	19	***MKLLVSLSLLASAAVTVLA***ADELGIEVTQAVNCERKTKS VSGPDVPQQQTEDVNGDMVHMHYKGTLQSTGDKFDAS YDRGTPLAFPLGTGRVIKGWDQGLLDMCIGEKRTLTIPP SLGYGDGGIGPIPGGATLIFETELVSIDGVETPEKIDYVSK AEEAASKATEKVAEKVADKIKEAAEVVKTVVADSDDA GQEHNEL
Mo-00804_80	307	18	***MKTSSIILAASLAATAVA***LPAGGGEPDRVVTITVPAPPTA ATAAPTWNWREGAVDSYPIHSSCNGTERLQLARALDET VSLARQARDHILRFGKTSSLYSKYFGNASTAEPVGWYH KLVGGDKAGLLFRCDDIDGNCHQEGWGGHWRGENATS ETVICPLSYTTRKSLEGLCGFGYTVAAGKLNTFWAGDL MHRIFHLEPVGEGVLEHYADSHAECLELAKSDPAKAAR NSHTLQYFALDVYAYDIALPGEGCAGKSPSASSDGGHST PSPIPASPPTQTSAAPTLLRAWIIGHVGNGRTKARLMLG
Mo-01842_14	245	18	***MKTSTSAVLAGLVAVATA***AVQLEVRYSDRMVDVGTLDL MEVTRNAIYAEPGNERSILTDRTHQAITRTCKSAVENTP DVSVQVKMTGAWGRTPGLKNNEMRDGLVASIFEALKQ VSDDTGYEVYSECRGLVWQESVAHVPEAACGRAAASG QTCDGPCRNAVASPGTTQCMKHDWGHRVPSVMRITAYI DDALQPDDLIFEFASTQNAQAGGCGIIGKIAGKLASFTIP VAGGLFSEGINILCAN
Mo-01560_1	319	17	***MLQTASLAAAALIGLLA***GPAPVLGASSKFCEGGSWSTLG KTGGNDRFRGEVDAPSGDFKVQGQFVEFMIDPATFAIYN YAWLGKENVGDMTGRQYTNIFASKVPDHRGANLTSKIS LEIKEEVIKISRTGNGVSMRISAKDCAQGGIFQMEPERAD NTSTRIVHTLADGAFQYDNPIFRSNLGKFMGSECVSEAG PAAEACVQVRPRVNIGAAGRPKMVLRDSAQVASRLPQP ECGPDFSNNLGIGGETRDYCGGMSIWDVASGGRMGMV TGEDSVEVSSPPDVCVSDCFPGDHPATGELAVLGFPDPV PEEVKLKPFM
Mo-02058_16	226	29	***MANGNMFRMVTWGLTIPIVLFYVLSLVGC***ISNSPGIPDIF LLKLTTPETETPYQVRIGYFGLCAGSDDAKLSCLPSFSLG RAPGTTVFNTTLPAELTRLVELGHEVQVKVIFFVPLLLAS VLFFVSTTTVFLWRNVPQISPMAATVAFLMHSLALGLLL AAATAVSMVVNALHFAVVEITRGQNEINVEGGKALQTF QWLTVIFSCVWQVCILKRYRSDMAGGKRI

Mo-02253_29	250	21	***MHISRITQFLAVLATATPILA***QEAVAAADFDVVERRYVG<br>GGVAMAGLAARNENNFFAKQLVAKDEDSGDVIIRDTSD<br>EGESGIEARDPRKKKNKKNKKKKGKKGKKKNKKAKAK<br>NAKAAQAGKANAAAAAAAPAADANKAVAAGAKAGA<br>GAGAAGAGAADAKKAGAGAADAKAGAGAAGVGAAD<br>AKAAGTGAAATKRDVDEVDAQTEEDHALETREPKRRK<br>GGKRKGGKKANGKKNKRDLDEVTESAEEP
Mo-00867_3	184	20	***MKVLQLLFVTLLTNAFMVAA***NNGGDDDCAAAEEKCIR<br>DCQAQGVKGIPDVVGRRGPLVGQSSQSRGLLFDSPAQC<br>GELVFDSTVISMRYKSAGKQQIKRAVEESAHPKPPFVCN<br>QRNREASRAVWVTLSNDGQLIARLAGTNASASVATTTD<br>IAALWRDVKVQRGRDLGHGLGLNKGILIINQR
Mo-00799_10	137	28	***MRPSNIFTTTTTTLACILGVAVAAPSDG***ICTTVHGGQTFH<br>NELRCIAEFYEHGDISNYFCHPKSDVKPGGVIKCVYRDH<br>PIVVKTGPGFNWKSTAGEMEQLKQKGVELKLLRRCMSE<br>RRNPYPPYSWNAGYGIEQAA
Mo-01957_5	119	22	***MHFKTLIATAITGSALFPCVLA***VHECFTTIHAKTPFRSSY<br>SPEYYKASLLQMRPGEDYYFKNPSPFEGCLVKFDEECTQ<br>LIWERLFDFPETTACPPADAKVDVPTRKEVAPPGTKTKL<br>N
Mo-02144_4	280	18	***MLLHSFIFSLALSGSAIA***GKPSSSSSDLPKMIPVKYMYRV<br>DLRPPKLIKKYCDDFICRDQLKDTPETPYGISFTSRFSAD<br>KFLKNNFPLASIHIYIIELLDDDPNDKDFRPHMVAAEAEA<br>VAYLRNPKAREPLSKTEFPPDGKFRTHAEDKKMVAAMK<br>KAISTGLRGIPSDTIASKKDQQRVLESPWRKVEGWVVPK<br>NYEHPMIHWSRDFVAYNPGPQVISGVCRFFENCGRKAV<br>GKQPKPAPVPLIPMTDTVVSPSARRIAPAQGSSILRDDYW<br>LYPAT
Mo-01609_4	83	20	***MKTYTAILVAVLAAVTTITA***APATSPVEQPFANIVAREQC<br>FHPGECGWTRSGQCEYHCDGYGGFMYMQGCGWGRKR<br>CCCAKKT
Mo-01389_1	82	19	***MKFSAIYLAALTLVSGAAA***ADPKNCKFNVLNGSGINVAN<br>GCCDVTKKQVTVSPFTVSCTESCGLAIVSGGDPTFSLQN<br>AGRC
Mo-00620_5	203	17	***MQFKNMLLLASATSVLA***APTTLEAPVANVDAAVPAIEFG<br>TAGHIETQAAAEEPEHSLVERQAISGAIIGALTPILQDLAT<br>KAVNEAIKQAGIFIKDLRNWTAAREAFTQATVREMWYS<br>NPDPAKYPAVVCYNKGYHLERPEGIAGWTKVELKTGFL<br>HTDYDCMYMEGNNNFYTWSDGGFINYAFGIDRNRCEF<br>DRSTGDILCR
Mo-00803_1	87	20	***MQFKSLFAFAALTLIPAVSA***LGCEVLISKKSGGDGPVKSS<br>CIPKSGSKVIVINGKTVTVSADGSCKFSSKDLDPSLAMKF<br>EGDCIGI
Mo-00464_1	290	19	***MKYTSAILISAFAATNVFA***HGVVTEVQGANGVTLPGLTA<br>IDGTPRDCPNPGCGSEADTAIIRDRELGTSRASALGRTQG<br>GGPVDAAKMIELFMDGASVNKSDVVAARERHAANLAR

			RATLLPRAGGGTSTPKGTEETGVKAATGIAATKGLPTTNDDGTINIVFHQVNQDGAGPLTADIDSTSGGQDVSAFQKAKITTNVPGLGIAGLSAAQTMDFPVAVQMPAGATCSGSVGGANNVCIARLRNAAVTGPFGGSVAFTQSPAARKRAIEYNLAKRRFARSLATDEEDDE
Mo-00985_1	103	19	***MRATTAFQVIAFLAVGAAA***APTGLVQARAVDSLAVETDDIASSAGPRADDNGAASAGLKVKRQREYWCPNQVCAKTFATQEERDHHIANTVHPTNSKRDVLLQ
Mo-00195_3	144	18	***MRFIFPLVIAGLAHVALA***APVRSSESAHSIVSRGTATEVYGVSTGEVLSKRAAVIAAGAYGDDDDSDDGWGESTAQRNRRKQHEKEARLLKAEEKKRLKEEQAKKRAAQKAEREAQREAKAAMKKQAGEAGQKSGCCGLLPGGN
Mo-01528_32	294	20	***MFSKALMLALAASPLVAAHG***KVAVVTGDQGGNGTALGILGGVVPGPGRNSVTEKDTTVFSKKQAGTNIMSDGLGKTKGNGENTPEMLSLAMAQSGDTLPQVSANGGTLSGTYHIVTTDGAGPIKAVLDPTGTGKFSEGMMLEVTTQVPGTNGNIKPPKKGKRSFLGEIWERSLDALEARGLVKRASNVNTDHPMSFAIPAGVKCTGEMAGQKNVCLVKIANENKAGPFGGCIAMQIAGTGGAAAGNATAPATTPAAAPAAADVKAADADDEAGDDKKKDKKNKKNAKNAKRFTA
Mo-01537_25	147	20	***MKVSLSIAAFLGALLATAAP***ADLSGAVADLSNSALPPVLTSSAADKVEPPTEKSVSGSKTGPPVCGTCHEGTKQCCGLLVATGAYGCYNQTCTVAQQQKTGPKNQALLQGLRGGGGNATRAADNSTVHTVNLTTVLDKVTLSPLALP
**Mo-01127_86**	**248**	**17**	***MLVALFSVALFASSALC***QDAAHQWQAPGSGDRRSPCPLLNSLANHGYLPRNGKDISVDALIDGMHAGLNLRDDAKLFFRLQGNKAVTASSTGSKDTFHLNDLNKHDLIEHDASLSRADLFFGDNWSFNQTIFDETKSHWPSPTISVRDAARALAARQKTAQAVNPKFNLPLDGHTNSLGQTAMYLGLFGNYDNGNANRSWVEYFFENERLPYELGWKRRSDDDKIPATGILGLTVQVAVDYLAVKIGL
Mo-02022_5	304	23	***MPSQTLLPALLLLLLSSTTPAAA***SFNWDITAHGVVSGWRWEKPWPHDDSPLMQFDELCRASATFPARQYKLSDLDADPAARAYAPAVRDLATGRVYPGSWDGVNVKGPRRDVLLVDWARVPRLARGWIRAQLETEDGRSRHFFRVVEKQAEKMPGLGGGGSGVVVEGVVDSSQSLEGSRDEHKVLITAPGELYQFLPLWVAEGSKCEDAFKDLTGYVATRAKDTCVVAWPTDHSRPLLEQGSKDINFTIEARLVKETDDGRAARIFWEKHHQNMQRLHRRIDREERAASRKGVERDRAAPRDEL
Mo-00337_8	70	23	***MLFSESTIAVLLAMFAHLSLAVA***EIAEASVKVTGGGSHGRVVEERGPPAPKKTKTKHRPRIQRRQAGFRP
Mo-01695_7	161	22	***MFATRLVSATAVLMALFAGALA***ASLHLAGGDFRMGVIYPRQQARALQVFDKALGGAKAQEITQSNDEKRPFQLGGDTFMWLTANDDLSCTIQTDFKSAADRSCDNQKNACAELSNSKKIDAPVSECDRQNTECKAAAASATQTAFNALVSSN

			AEFDFFCDL
Mo-01499_3	113	16	***MAAILLGFIASSAVVA***LPAGIQAGPDHPDRVQRRGASSS AQRSGGDKSEDRPSLREIMKEPGWAWRIASLTPTGRELV KEAKIAQQERDEARRRAFEAGYSPDPRAGGTSVRA

Supplementary Table S2: *In silico* functional annotation of selected 54 candidate AvrPi54 proteins obtained by performing BLAST P against non-redundant protein database available at NCBI

**Protein ID**	**BLAST score**	**e-value**	**Functional annotation**
Mo-00195_3	366	$2E^{-33}$	Hypothetical protein
Mo-00288_9	480	$1E^{-46}$	Hypothetical protein
Mo-00337_8	154	$6E^{-09}$	Hypothetical protein
Mo-00464_1	1497	$1E^{-164}$	Hypothetical protein
Mo-00481_2	188	$9E^{-13}$	Hypothetical protein
Mo-00541_1	738	$1E^{-76}$	Hypothetical protein
Mo-00552_8	947	$1E^{-100}$	Hypothetical protein
Mo-00620_5	938	$2E^{-99}$	Hypothetical protein
Mo-00684_1	721	2E-74	Hypothetical protein
Mo-00799_10	349	$2E^{-31}$	Hypothetical protein
Mo-00803_1	427	$2E^{-40}$	Hypothetical protein
Mo-00804_80	1204	$1E^{-130}$	Hypothetical protein
Mo-00831_7	432	$4E^{-41}$	Hypothetical protein
Mo-00867_3	665	$5E^{-68}$	Hypothetical protein
Mo-00870_1	596	$4E^{-60}$	Hypothetical protein
Mo-00952_14	452	$3E^{-43}$	Hypothetical protein
Mo-00985_1	483	$5E^{-47}$	Hypothetical protein
Mo-01127_86	1145	$1E^{-123}$	Hypothetical protein
Mo-01300_5	677	$2E^{-69}$	Hypothetical protein
Mo-01348_1	509	$5E^{-50}$	Hypothetical protein
Mo-01361_2	321	$3E^{-28}$	Hypothetical protein
Mo-01389_1	351	$9E^{-32}$	Hypothetical protein
Mo-01492_64	303	$2E^{-103}$	Hypothetical protein
Mo-01499_3	406	$4E^{-38}$	Hypothetical protein
Mo-01514_2	538	$2E^{-53}$	Hypothetical protein
Mo-01528_32	538	$7E^{-53}$	Hypothetical protein
Mo-01537_25	542	$7E^{-54}$	Hypothetical protein
Mo-01560_1	1781	0	Hypothetical protein
Mo-01609_4	319	$6E^{-28}$	Hypothetical protein
Mo-01622_43	663	$7E^{-68}$	Hypothetical protein
Mo-01643_3	1184	$1E^{-128}$	Hypothetical protein
Mo-01695_7	545	$3E^{-54}$	Hypothetical protein
Mo-01702_12	513	$2E^{-50}$	Hypothetical protein
Mo-01702_7	478	$2E^{-46}$	Hypothetical protein
Mo-01702_7	478	$2E^{-46}$	Hypothetical protein
Mo-01702_9	287	$3E^{-24}$	Hypothetical protein

Mo-01733_6	390	$3E^{-36}$	Hypothetical protein
Mo-01809_3	289	$3E^{-24}$	Hypothetical protein
Mo-01842_14	1132	$1E^{-122}$	Hypothetical protein
Mo-01947_9	595	$4E^{-60}$	Hypothetical protein
Mo-01956_6	789	$2E^{-82}$	Hypothetical protein
Mo-01957_5	615	$3E^{-62}$	Hypothetical protein
Mo-01980_82	1127	$1E^{-121}$	Hypothetical protein
Mo-01992_3	489	$1E^{-47}$	Hypothetical protein
Mo-02022_5	1276	$1E^{-138}$	Hypothetical protein
Mo-02035_52	206	$8E^{-67}$	Hypothetical protein
Mo-02058_16	1022	$1E^{-109}$	Hypothetical protein
Mo-02144_4	1360	$1E^{-148}$	Hypothetical protein
Mo-02164_23	846	$4E^{-89}$	Hypothetical protein
Mo-02191_8	1254	$1E^{-136}$	Hypothetical protein
Mo-02196_3	374	$3E^{-34}$	Hypothetical protein
Mo-02196_64	679	$3E^{-69}$	Hypothetical protein
Mo-02253_29	310	$2E^{-26}$	Hypothetical protein
Mo-02266_3	235	$3E^{-18}$	Hypothetical protein

Supplementary Table S3: Various features of the selected candidate *AvrPi54* genes

Gene ID	General information					Result of BLASTN against EST database of NCBI		
	Chromosome containing the gene	Length of ORF (nucleotides)	No. of exons	Length of protein (amino acids)	Length of signal peptide (amino acids)	Bit score	E-vlue	Query coverage
Mo-01702_7	7	312	1	103	21	1057	0.0	74%
Mo-01127_86	2	747	3	248	17	623	$6e^{-65}$	78%
Mo-01947_9	4	462	1	153	19	848	0.0	100%
Mo-02191_8	1	804	3	267	21	733	0.0	54%

Supplementary Table S4. Details of descriptions about the structural features of modeled candidate AvrPi54 proteins

Protein ID	Number of α helix	Number of β sheets	Ramachandran Plot Analysis		
			Residues in favoured region (%)	Residues in allowed region (%)	Residues in outlier region (%)
Mo-01702_7	28	0	91.2	5.0	3.8
Mo-01127_86	12	2	93.0	5.7	1.3
Mo-01947_9	0	6	95.4	2.8	1.9
Mo-02191_8	6	2	89.2	7.1	9

Supplementary Table S5. Important parameters and energy values of candidate AvrPi54 protein models docked against Pi54 protein model

Protein complex	Score†	Area (Å^2)#	ACE (kCal/mol)‡	QMEAN Z score	QMEAN
Pi54 and Mo-01702_7	15230	2271.80	424.98	-6.648	0.198
Pi54 and Mo-01127_86	14496	1942.10	490.16	-7.626	0.090
Pi54 and Mo-01947_9	13708	1800.50	-139.17	-6.260	0.237
Pi54 and Mo-02191_8	16186	2264.70	224.78	-7.682	0.080

†Geometric shape complementarity score #Atomic Contact Area ‡Atomic Contact Energy

Supplementary Table S6: List of amino acids present in different domains of Pi54 protein involved in interaction with candidate AvrPi54 protein

	**Interacting with 01947_9_complete**	**Interacting with 01947_9_truncated**
Amino acids of LRR domain of Pi54 protein	Leucine-224<br>Serine-225<br>Isoleucine-226<br>Phenylalanine-228	Leucine-221<br>Glutamate-222<br>Asparagine-223<br>Leucine-224<br>Serine-225<br>Isoleucine-226<br>Serine-227<br>Phenylalanine-228<br>Glutamate-231<br>Lysine-249
Amino acids of Zinc finger (Zn-fin) or near Zn-fin domain of Pi54 protein	None	Isoleucine-199<br>Leucine-200<br>Tryptophan-201<br>Methionine-202<br>Asparagines-203<br>Asparagines-204

Amino acids of NBS domain of Pi54 protein	Lysine-121<br>Glutamate-124<br>Serine-126<br>Glycine-127<br>Valine-128	Alanine-123<br>Glutamate-124<br>Serine-126<br>Glycine-127<br>Valine-128<br>Aspertate-129

Supplementary Table S7: Fisher's t-test for the comparison of the equality of sample means of infiltered constructs in agroinfiltration assays using OriginPro software

Constructs	Mean Diff (mm)	SEM (mm)	t-value	Probability	Level of Significance (α)	Sig
AvrPi54 Vs. Pi54+AvrPi54	-132.69	2.684	-49.420	1.83E-^{161}	0.05	1
Pi54 Vs. Pi54+AvrPi54	-132.707	2.684	-49.427	1.76E-^{161}	0.05	1
Pi54 Vs. AvrPi54	-0.016	2.684	-0.006	0.99508	0.05	0
Empty vector Vs. Pi54+AvrPi54	-154.395	2.684	-57.505	2.80E-^{182}	0.05	1
Empty vector Vs. AvrPi54	-21.705	2.684	-8.084	9.89E-^{15}	0.05	1
Empty vector Vs. Pi54	-21.688	2.684	-8.077	1.03E-^{14}	0.05	1

SEM: Standard error of mean

Sig = 1 indicates that the difference between the means is significant at the 0.05 level of significance while Sig = 0 shows that the difference between the means is non-significant at the 0.05 level of significance

Supplementary Table S8: Phenotyping data of TP-309 and TP-2 inoculated with *M. oryzae* isolates MG-79 and MG-79_AvrPi54[1]

	MG-79_ $AvrPi54_{Tr1}$	MG-79_control	MG-79_ $AvrPi54_{Tr2}$	MG-79_control	MG-79_ $AvrPi54_{Tr3}$	MG-79_control
TP-2_Pt 1	2	4	3	4	1	3
TP-2_Pt 2	2	4	2	4	1	4
TP-2_Pt 3	1	4	2	5	1	4
TP-2_Pt 4	2	5	2	5	0	5
TP-2_Pt 5	1	3	2	3	2	4
TP-2_Pt 6	1	5	1	4	1	5
TP-2_Pt 7	3	5	3	4	2	4
TP-2_Pt 8	0	3	1	3	0	4
TP-2_Pt 9	0	2	1	4	1	4
TP-2_Pt 10	2	4	2	5	0	5
TP-2_Pt 11	2	4	2	4	2	4
TP-2_Pt 12	3	3	0	3	0	2
TP-2_Pt 13	1	3	1	4	2	3
TP-2_Pt 14	1	4	0	2	0	2
TP-2_Pt 15	1	5	1	5	3	5
TP-2_Pt 16	1	3	1	3	1	4
TP-2_Pt 17	2	2	2	2	2	4
TP-2_Pt 18	2	4	3	4	1	4
TP-2_Pt 19	1	4	2	4	2	5
TP-2_Pt 20	3	4	3	4	0	5
TP-2_Pt 21	2	3	2	4	2	4
TP-2_Pt 22	2	5	1	3	2	3
TP-2_Pt 23	2	3	2	4	2	4
TP-2_Pt 24	3	5	1	5	2	5
TP-2_Pt 25	1	4	1	4	1	4
TP-2_Pt 26	1	3	0	3	1	5
TP-2_Pt 27	2	4	2	5	0	4
TP-2_Pt 28	4	5	2	5	1	5
TP-2_Pt 29	3	5	2	4	2	4
TP-2_Pt 30	1	3	1	3	1	4

	MG-79_ $AvrPi54_{Tr1}$	MG-79_control	MG-79_ $AvrPi54_{Tr2}$	MG-79_control	MG-79_ $AvrPi54_{Tr3}$	MG-79_control
TP-309_Pt 1	4	3	5	4	5	2
TP-309_Pt 2	4	4	4	4	5	3
TP-309_Pt 3	3	5	5	4	4	5
TP-309_Pt 4	5	4	5	5	5	4
TP-309_Pt 5	3	4	4	4	5	4
TP-309_Pt 6	5	5	4	3	4	5
TP-309_Pt 7	5	4	3	4	3	5
TP-309_Pt 8	4	3	5	5	3	5

TP-309_Pt 9	3	5	3	5	3	5
TP-309_Pt 10	2	4	2	5	4	4
TP-309_Pt 11	3	4	4	4	4	3
TP-309_Pt 12	3	3	5	4	5	4
TP-309_Pt 13	4	4	4	5	4	4
TP-309_Pt 14	5	2	5	3	3	5
TP-309_Pt 15	5	3	5	4	5	5
TP-309_Pt 16	5	2	3	5	4	4
TP-309_Pt 17	4	4	3	3	4	5
TP-309_Pt 18	4	4	4	4	3	4
TP-309_Pt 19	5	4	5	4	4	4
TP-309_Pt 20	3	3	5	2	3	4
TP-309_Pt 21	4	5	5	4	4	4
TP-309_Pt 22	5	5	4	5	5	3
TP-309_Pt 23	3	4	4	5	4	4
TP-309_Pt 24	5	5	4	3	3	5
TP-309_Pt 25	4	5	4	4	5	4
TP-309_Pt 26	3	4	5	4	4	3
TP-309_Pt 27	4	5	3	5	3	4
TP-309_Pt 28	5	4	3	3	5	3
TP-309_Pt 29	3	3	5	4	5	4
TP-309_Pt 30	4	5	4	5	4	5
	MG-79_ AvrPi54$_{Tr1}$	MG-79_control	MG-79_ AvrPi54$_{Tr2}$	MG-79_control	MG-79_ AvrPi54$_{Tr3}$	MG-79_control
Tetep_Pt1	1	1	0	1	1	0
Tetep_Pt2	0	0	0	1	0	0
Tetep_Pt3	0	0	0	0	0	1
Tetep_Pt4	1	0	0	0	1	0
Tetep_Pt5	0	0	1	0	1	2
Tetep_Pt6	0	1	1	1	1	1
Tetep_Pt7	1	0	0	0	0	1
Tetep_Pt8	0	1	0	0	1	1
Tetep_Pt9	1	0	1	1	0	0
	MG-79_ AvrPi54$_{Tr1}$	MG-79_control	MG-79_ AvrPi54$_{Tr2}$	MG-79_control	MG-79_ AvrPi54$_{Tr3}$	MG-79_control
HR-12_Pt1	4	5	3	4	4	5
HR-12_Pt2	5	5	4	5	3	4
HR-12_Pt3	5	5	4	5	5	5
HR-12_Pt4	4	4	3	5	5	5
HR-12_Pt5	5	4	5	4	4	5
HR-12_Pt6	4	5	5	4	5	5
HR-12_Pt7	4	5	5	5	4	4
HR-12_Pt8	4	4	4	5	5	4
HR-12_Pt9	5	5	5	4	5	5

TP-309: Non-transgenic rice line (do not contain dominant Pi54 gene)

TP-2: Transgenic rice line (contain dominant Pi54 gene in TP-309 background)

Mg-79: Non-transgenic *M. oryzae* isolate (do not contain dominant *AvrPi54* gene)

MG-79_AvrPi54: Transgenic *M. oryzae* isolate (contain dominant *AvrPi54* gene in TP-309 background)

Pt: Represent individual plants

Tr: Represents individual transformants

[1] Disease reactions were recorded after 7 days of inoculation on a 0-5 disease assessment scale (Bonman et al. 1986) where a score of '0' represents no evidence of infection, while the score of '5' represents typical-spindle shaped blast lesions capable of sporulation of more than 3 mm in diameter and about half of 1-2 leaf blades killed by coalescence of lesions.

Supplementary Table S9: Details of primers used in this study. The restriction sites included in the primers are marked in bold

Purpose	Primer ID	Length (bp)	Sequence (5′-3′)	Tm (°C)	Product size (bp)
Yeast-2-Hybrid analysis	AD_EcoRI_Avr_F	34	GGAGGCCAGT**GAATTC**ATGCAGTTCACCGCCACC	69.2	494
	AD_BamHI_Avr_R	35	CGAGCTCGAT**GGATCC**CTAGCAGCCATAGGTGAGG	70.3	
	AD_EcoRI_Avr_tr_F	34	GGAGGCCAGT**GAATTC**GCCCCCGTTGAGGTCGCC	72.9	437
	AD_BamHI_Avr_tr_R	41	CGAGCTCGAT**GGATCC**CTAGCAGCCATAGGTGAGGATCAAA	70.5	
	BD_EcoRI_Res_F	36	AAAAA**GAATTC**ATGTCAGAGCTTCAATCACTGCCAG	62.2	1005
	BD_PstI_Res_R	36	GGTTT**CTGCAG**TTATGATGGTTCTTTAAAATTGGGG	62.2	
	BD_EcoRI_Avr_F	34	CATGGAGGCC**GAATTC**ATGCAGTTCACCGCCACC	69.2	494
	BD_BamHI_Avr_R	35	GCAGGTCGAC**GGATCC**CTAGCAGCCATAGGTGAGG	71.5	
	BD_EcoRI_Avr_tr_F	34	CATGGAGGCC**GAATTC**GCCCCCGTTGAGGTCGCC	72.9	437
	BD_BamHI_Avr_tr_R	41	GCAGGTCGAC**GGATCC**CTAGCAGCCATAGGTGAGGATCAAA	71.5	
	AD_EcoRI_Res_F	36	AAAAA**GAATTC**ATGTCAGAGCTTCAATCACTGCCAG	62.2	1005
	AD_BamHI_Res_R	36	GGTTT**GGATCC**TTATGATGGTTCTTTAAAATTGGGG	62.2	
Overlap Extension PCR	BamHI_CaMV35S_F	35	AAA**GGATCC**AGATTAGCCTTTTCAATTTCAGAAAG	51.7	870
	XbaI_CaMV35S_R	50	ATATTCCGTCAACTTTATAC**TCTAGA**CGTGTTCTCTCCAAATGAAATGAA	52.3	
	XbaI_AvrPi54_F	51	TTTCATTTGGAGAGAACACG**TCTAGA**GTATAAAGTTGACGGAATATCC	54.4	1310

			CTC		
	XmaI_AvrPi54_R	49	TGCCAAATGTTTGAACGATC**CCCGGG**TCGTTGGGAATTGGGTTTTACAC	53.5	
	XmaI_NOS_F	53	GTAAAACCCAATTCCCAACGA**CCCGGG**GATCGTTCAAACATTTGGCAATAAAG	53.2	289
	EcoRI_NOS_R	32	CCC**GAATTC**GATCTAGTAACATAGATGACACC	51.7	
Semiquantitive Reverse transcriptase PCR	sqRT_AvrPi54_F	22	CCGCCAGGATATGCAGTATAAG	62.0	102
	sqRT_AvrPi54_R	22	CCATAGGTGAGGATCAAACCAG	62.0	
	MgActin_F	21	AGCGTGGTATCCTCACTTTGC	62.0	175
	MgActin_R	22	ATCTTCTCTCGGTTGGACTTGG	62.0	

Supplementary Table S10: Media and solutions used for protoplast preparation, transformation and regeneration of *M. oryzae*

**Media and Solutions**	**Ingredients required with quantity**	**Final volume (mL)**
Complete media	6 g of yeast extract, 6 g of casamino acid, 10 g of sucrose and distilled water‡	1000
TB3 Broth	3g/L Yeast extract, 3g/L casamino acid, 10g/L Glucose, 200g/L sucrose and distilled water‡	500
TB3 Agar	TB3 broth, 8g/L agar, 250 µg/mL of sulfonyl urea and distilled water‡	100
Washing solution	200g/L sucrose and distilled water‡	1000
Protoplasting solution	200g/L sucrose, 250 mg lysing enzyme and distilled water‡	20
2X STC†	40 g of sucrose, 10 mL of 50mM Tris-HCl (pH8.0), 1.47 g of $CaCl_2$ and distilled water‡	100
1X STC†	2X STC and distilled water‡	100
2X PEG†	4 g PEG 800 and distilled water‡	5
PTC	0.5 mL of 2X STC mixed with 0.5 mL of 2X PEG	1

† 'X' denotes concentration

‡ Distilled water was used as solvent to dissolve the solutes and also for final volume makeup

STC: Sucrose-Tris-Calcium chloride medium; PEG: Polyethylene glycol; PTC: PEG-Tris-Calcium chloride medium

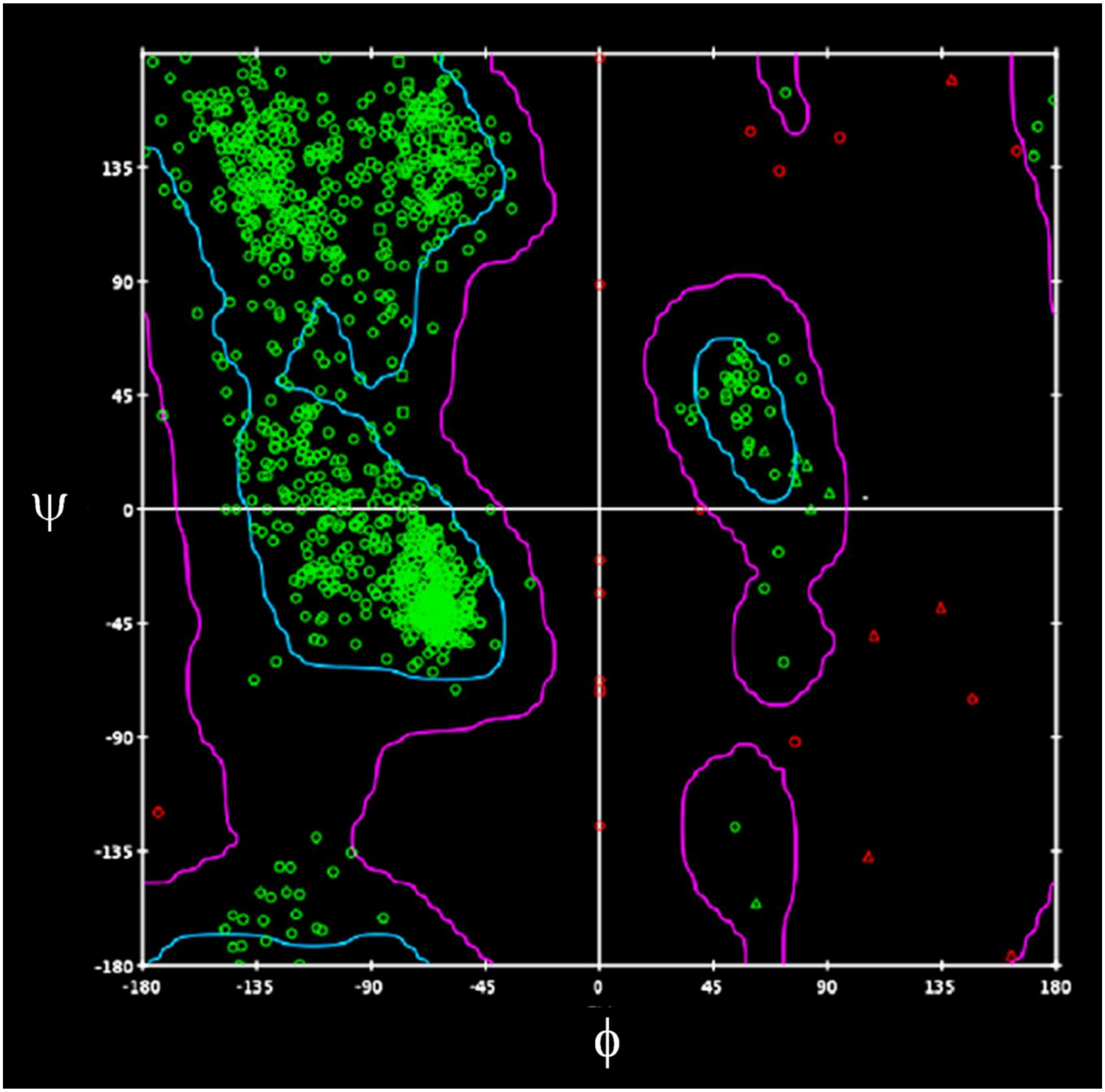

Supplementary Figure S1: Ramachandran plot of full-length AvrPi54 protein.

Out of 153 amino acids of the full-length protein, 68.0% (102 amino acids) were in favoured region, 21.3% (16 amino acids) were in allowed region and rest 10.7% (19 amino acids) in disallowed region. The azure lines mark the border of favoured and allowed region, while the pink line mark the border of allowed and disallowed region. The amino acids in disallowed region are marked in red and rests (falling in favoured and allowed region) are marked in green. The triangles represent the proline residues while circles represent residues other than proline.

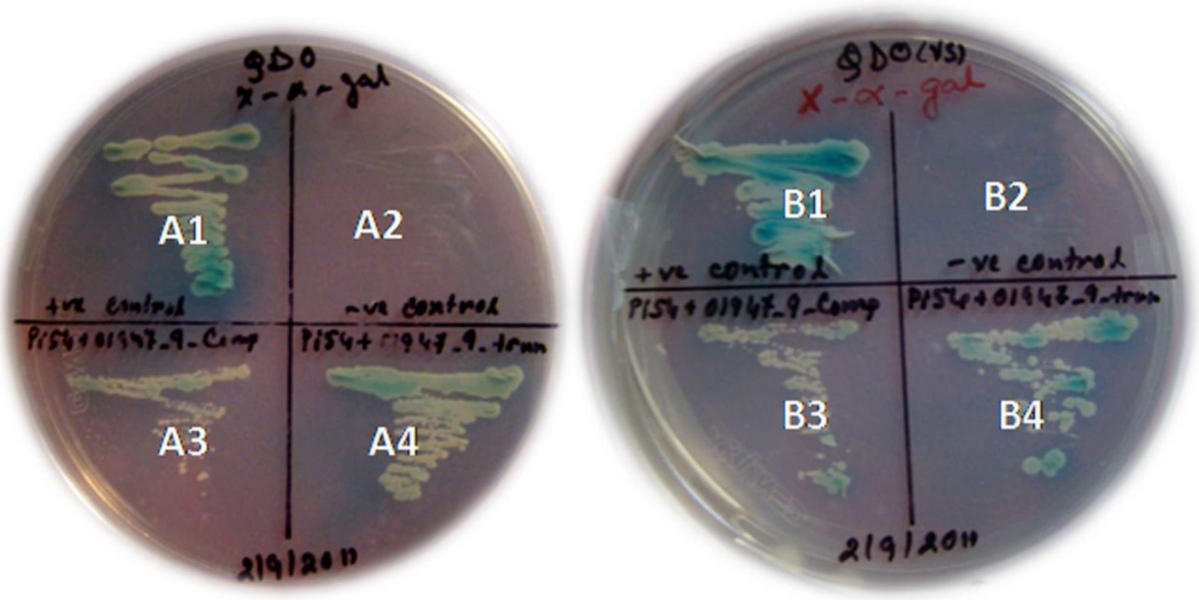

Supplementary Figure S2: Vector swapping experiment to confirm the authenticity of yeast-2-hybrid analysis

Plate on the left shows initial fusion where A1 and A2 are positive and negative controls for interaction respectively, A3 show interaction of BD::Pi54 with AD::AvrPi54_comp and A4 shows interaction of BD::Pi54 with AD::AvrPi54_trunc. Plate on the right shows reverse fusion where B1 and B2 are positive and negative controls for interaction respectively, B3 show interaction of AD::Pi54 with BD::AvrPi54_comp and B4 shows interaction of AD::Pi54 with BD::AvrPi54_trunc.

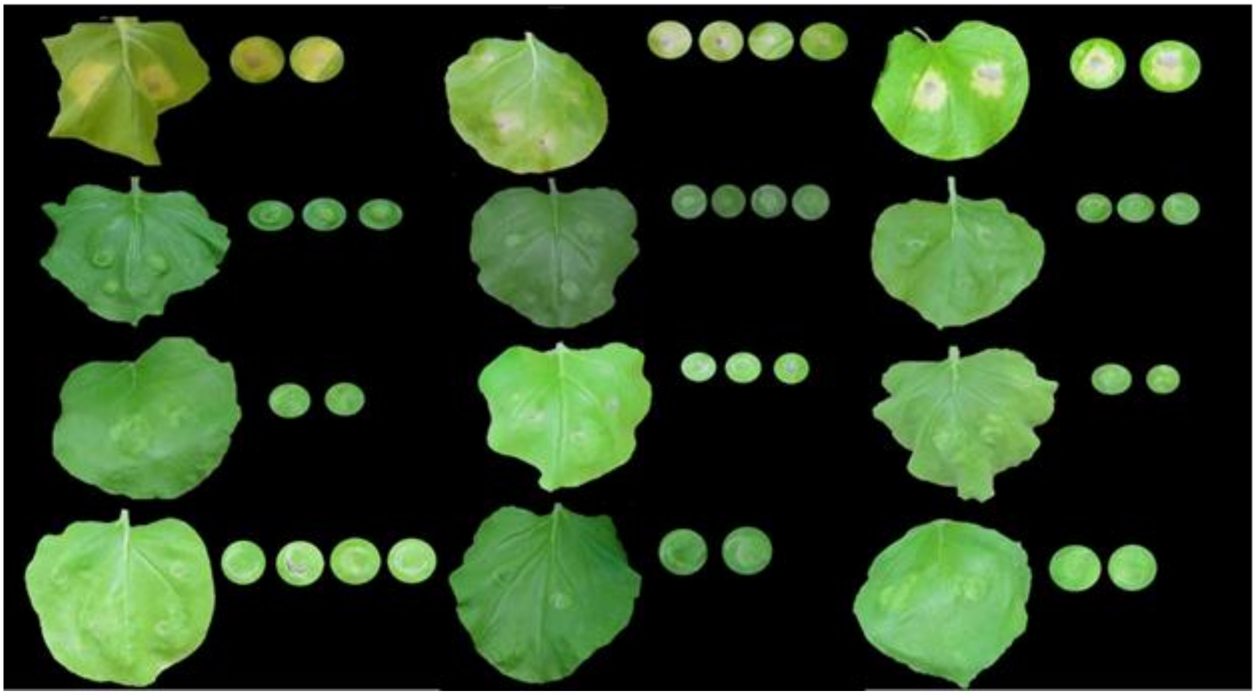

Supplementary Figure S3: Interaction of AvrP54 and Pi54 proteins using agroinfiltration assay

Interaction of *AvrPi54* gene product with its counterpart gene product Pi54 through Agroinfiltration assays. Top most upper row of this panel shows co-expression and interaction of both genes, *Pi54* and *AvrPi54* (pCAMBIA:Pi54+pCAMBIA:AvrPi54) in leaves of *N. benthamiana*; second row from top for *AvrPi54* gene alone (pCAMBIA:AvrPi54), third row from top for *Pi54* gene alone (pCAMBIA:Pi54) and last row from top for empty vector used as a negative control pCAMBIA1305.1 (without any insert) represent infiltration assays in leaves of *N. benthamiana*. In this panel, the inoculated spots were cut out from the respective leaves for better visibility.

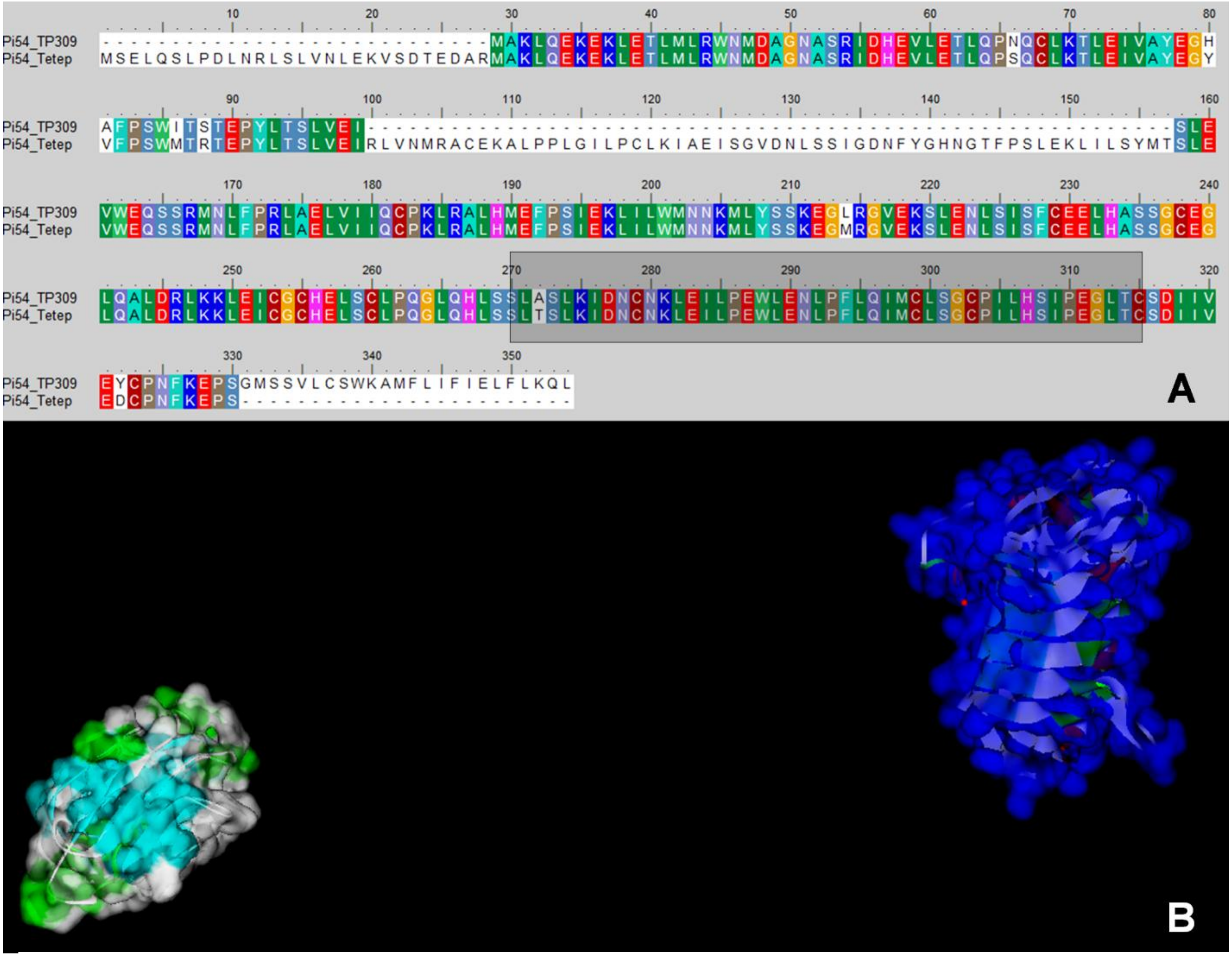

Supplementary Figure S4: Interaction potential analysis of susceptible allele encoded protein of *Pi54* gene with candidate *AvrPi54* gene.

Alignment of protein sequences encoded by of resistant and susceptible alleles of *Pi54* gene (output of bioedit software). Two large deletions are visible in the N-terminal region of susceptible allele encoded protein (Pi54_TP-309) as it is aligned against resistant allele encoded protein (Pi54_TP-309). Intact LRR domain at the C-terminal region of both the proteins is marked transparent inside black box (A). Docking result of AvrPi54 protein against Pi54_TP-309 protein encoded by susceptible allele of *Pi54* gene obtained from susceptible rice line, TP-309. The two proteins remained distant enough after completion of docking to make any physical interaction possible. Pi54_TP-309 is marked in blue (B).

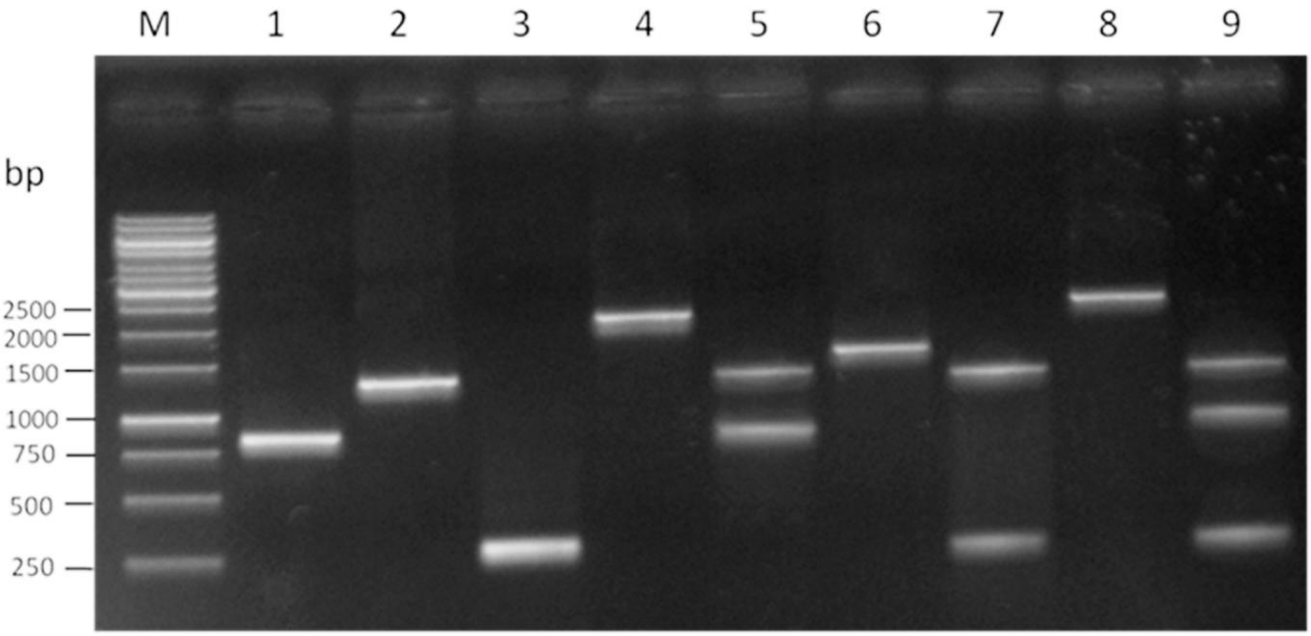

Supplementary Figure S5: Construction of *AvrPi54* expression cassette using Overlap Extension PCR (OE-PCR)

CaMV35S promoter_(870 bp, lane 1), *AvrPi54* gene (1310 b, lane 2) and Nos terminator (289 bp, lane 3) were amplified from specific target sites. CaMV35S and *AvrPi54* were joined using OE-PCR to obtain 2134 bp amplicon (lane 4) having *XbaI* restriction site in between. Subsequent *XbaI* digesition of the amplicon released original fragments (lane 5). Similarly, *AvrPi54* and Nos were joined by OE-PCR to obtain 1552 bp amplicon (lane 6) having *XmaI* restriction site in between. Subsequent *XmaI* digesition of the amplicon released original fragments (lane 7). Finally, these two amplicons were joined with each-other by OE-PCR in order to construct the whole expression cassette containing CaMV35S, *AvrPi54* and Nos (2376 bp, lane 8). Double digestion of the 2376 bp amplicon with *XbaI* and *XmaI* yielded the original fragments (lane 9) confirming successful expression cassette construction.
